# Mining of Marburg Virus Proteome for Designing an Epitope-Based Vaccine

**DOI:** 10.3389/fimmu.2022.907481

**Published:** 2022-07-15

**Authors:** Mohamed A. Soltan, Waleed K. Abdulsahib, Mahmoud Amer, Ahmed M. Refaat, Alaa A. Bagalagel, Reem M. Diri, Sarah Albogami, Eman Fayad, Refaat A. Eid, Sherin M. A. Sharaf, Sameh S. Elhady, Khaled M. Darwish, Muhammad Alaa Eldeen

**Affiliations:** ^1^ Department of Microbiology and Immunology, Faculty of Pharmacy, Sinai University, Ismailia, Egypt; ^2^ Department of pharmacology and Toxicology, College of Pharmacy, Al- Farahidi University, Baghdad, Iraq; ^3^ Internal Medicine Department, Faculty of Medicine, Zagazig University, Zagazig, Egypt; ^4^ Zoology Department, Faculty of Science, Minia University, El-Minia, Egypt; ^5^ Department of Pharmacy Practice, Faculty of Pharmacy, King Abdulaziz University, Jeddah, Saudi Arabia; ^6^ Department of Biotechnology, College of Science, Taif University, Taif, Saudi Arabia; ^7^ Department of Pathology, College of Medicine, King Khalid University, Abha, Saudi Arabia; ^8^ Department of Microbiology, Egyptian Drug Authority (EDA), Giza, Egypt; ^9^ Department of Natural Products, Faculty of Pharmacy, King Abdulaziz University, Jeddah, Saudi Arabia; ^10^ Department of Medicinal Chemistry, Faculty of Pharmacy, Suez Canal University, Ismailia, Egypt; ^11^ Cell Biology, Histology and Genetics Division, Zoology Department, Faculty of Science, Zagazig University, Zagazig, Egypt

**Keywords:** Marburg virus, immunoinformatics, epitope mapping, multitope vaccine, health care

## Abstract

Marburg virus (MARV) is one of the most harmful zoonotic viruses with deadly effects on both humans and nonhuman primates. Because of its severe outbreaks with a high rate of fatality, the world health organization put it as a risk group 4 pathogen and focused on the urgent need for the development of effective solutions against that virus. However, up to date, there is no effective vaccine against MARV in the market. In the current study, the complete proteome of MARV (seven proteins) was analyzed for the antigenicity score and the virulence or physiological role of each protein where we nominated envelope glycoprotein (Gp), Transcriptional activator (VP30), and membrane-associated protein (VP24) as the candidates for epitope prediction. Following that, a vaccine construct was designed based on CTL, HTL, and BCL epitopes of the selected protein candidates and to finalize the vaccine construct, several amino acid linkers, β-defensin adjuvant, and PADRE peptides were incorporated. The generated potential vaccine was assessed computationally for several properties such as antigenicity, allergenicity, stability, and other structural features where the outcomes of these assessments nominated this potential vaccine to be validated for its binding affinity with two molecular targets TLR-8 and TLR-4. The binding score and the stability of the vaccine-receptor complex, which was deeply studied through molecular docking-coupled dynamics simulation, supported the selection of our designed vaccine as a putative solution for MARV that should be validated through future wet-lab experiments. Here, we describe the computational approach for designing and analysis of this potential vaccine.

## Introduction

Marburg virus (MARV) is a notorious pathogen that belongs to *Filoviridae* family. It was first discovered in West Germany in the year of 1967 (1). It was found that the Egyptian fruit bat (*Rousettus aegyptiacus*) acts as a reservoir for MARV where primary human infection occurs through exposure to the viral reservoir, then transmission between people occurs *via* body fluids (2). MARV pathogenesis investigation showed that it causes life-threatening hemorrhagic fever which was very similar to that of the Ebola virus (EBOV) as both fevers are characterized by a severe inflammatory reaction in addition to systemic hemorrhaging (3). Since its early discovery, MARV has shown several successive waves of outbreaks. Outbreaks with a large number of cases were shown in Congo between 1998 and 2000 and in Angola between 2004 and 2005 with fatality rates of 83% (4) and 90% (5) respectively. The last few years showed continuous outbreaks of MARV where Uganda (6) and Guinea suffered from these outbreaks with a 100% fatality rate.

The MARV proteome and genome analysis showed seven basic proteins in addition to a negative-stranded linear RNA genome that sized approximately 19 kb (7). Most MARV proteins are multifunctional with major roles in viral replication and pathogenesis. The transcriptional activator VP30 was found to be involved in nucleocapsid maturation (8). The polymerase cofactor VP35 is an essential cofactor in the process of viral replication (9). Envelope glycoprotein has a major role in the interaction with the cellular receptors of the infected host (10). Membrane-associated protein VP24 plays a significant role in nucleocapsid and viral matrix formation (11). MARV nucleoprotein encapsidates the viral genome by oligomerization (12) while RNA-directed RNA polymerase is responsible for replicating this genome. Finally, matrix protein VP40 regulates the process of virion assembly and budding from infected cells (13).

The world health organization has put MARV on a list of an urgent need to find a solution for this deadly virus where the severe outbreaks and the high fatality rate have increased the importance of this call. Consequently, several techniques including vaccines based on viral vectors such as vesicular stomatitis virus (14), Adenovirus vectored vaccines (15), DNA plasmid vaccine (16), virus-like particles composed of several MARV proteins (17), and recombinant vaccine (18) have been adopted to generate an effective vaccine against this deadly virus. However, up to date, there is no approved vaccine or drug against MARV in the market.

In the last few years, the scientific community witnessed major development in the fields of bioinformatics, structural biology, and computational tools that were designed for the analysis of the growing data of several organisms’ genomes. More recently, a new field that studies the immunological data and the tools that were developed to handle these data has been named immunoinformatics (19). This novel approach was applied to develop a vaccine construct against many pathogens, starting from bacteria such as *Moraxella catarrhalis* (20) and *Escherichia coli* (21) to viruses such as Nipah virus (22) and fungi such as *Candida auris* (23) and Mucormycosis causing fungi (24). Compared to classical and single-epitope vaccines, multi-epitope vaccines have unique features in their design as they consist of multiple MHC-restricted epitopes in addition to B-cell epitopes that can be recognized by TCRs of multiple clones from various T and B cell subsets. Consequently, strong cellular and humoral immune responses can be generated simultaneously. Moreover, multi-epitope vaccines incorporate some components with adjuvant capacity that can enhance the immunogenicity and long-lasting immune responses and reduce unwanted components that can trigger either pathological immune responses or adverse effects (25). Due to these several advantages, many trials have been performed to explore the efficacy of this new form of vaccine, where a significant activation at humoral and cellular arms of the immune system was observed against several tested pathogens such as *E. coli* (26), *Salmonella Typhimurium* and *Shigella flexneri* (27), and HIV-1 infection (28).

In the current study, we applied the immunoinformatics approach to generate a potential vaccine construct against MARV. The viral whole proteome was firstly analyzed for antigenicity and virulence of each protein then the candidate proteins were extracted for B and T cell epitope prediction. Finally, the top-ranking epitopes of each candidate were selected to construct a chimeric epitope potential vaccine that was assessed computationally for its structural, immunological, and chemical characteristics to be nominated as a putative solution against MARV.

## Materials and Methods

**Figure 1:**
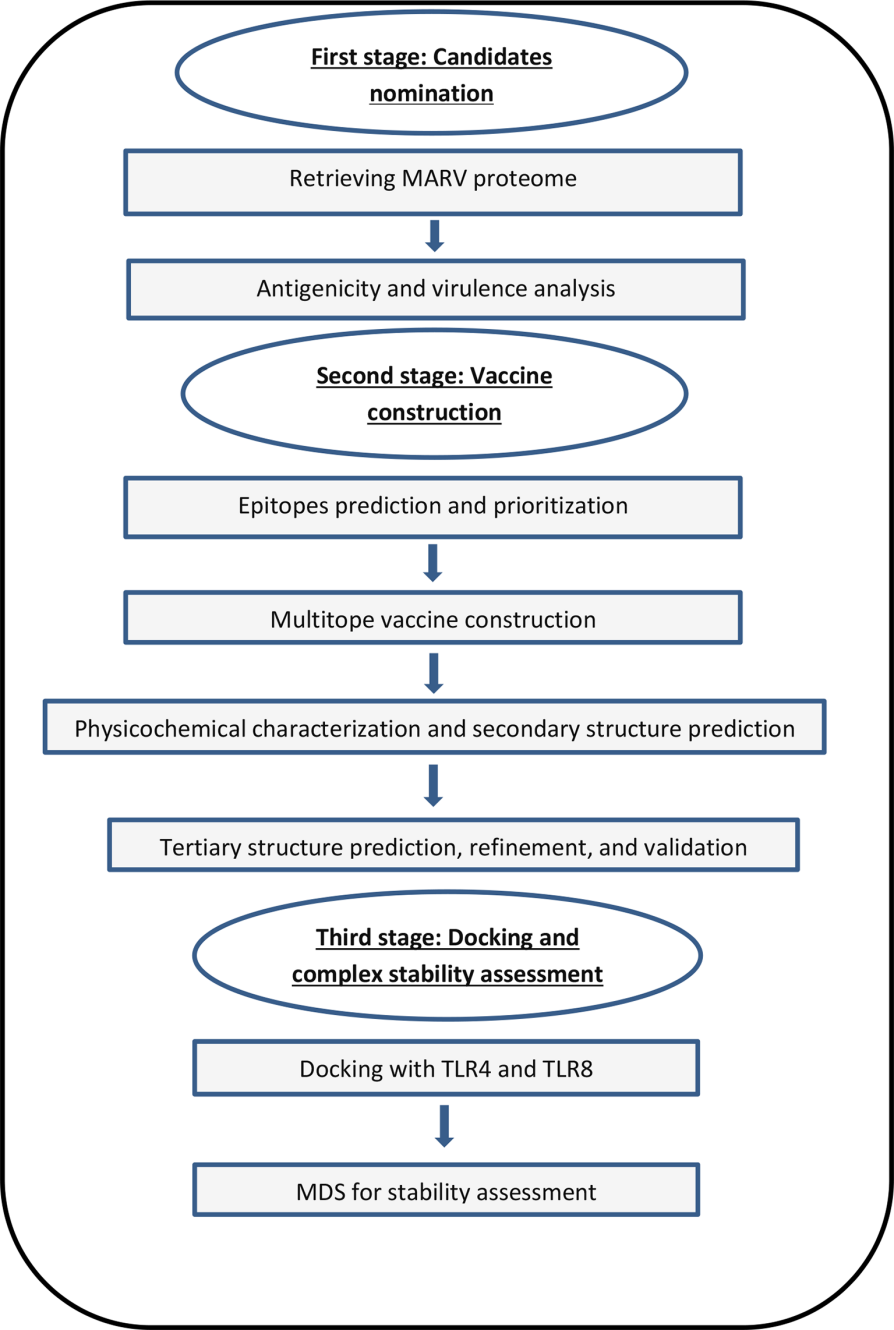
A graph showing the applied strategy of the current study for designing and assessment of a potential vaccine against MARV.

The flow of work of basic stages that were applied in the current study is illustrated in **Figure 1**.

### Selecting Vaccine Candidates

UniProt database was used to retrieve Lake Victoria marburgvirus (strain Ravn-87) standard proteome which had the proteome Id (UP000008239). The analysis of the whole proteome was performed to identify proteins with an antigenicity score that exceeds 0.5 besides a necessary physiological role or virulent role for MARV to allow nominating them as the vaccine candidates of our study. Vaxigen v2.0 was used to calculate the antigenicity score (29). Upon using UniProt for MARV proteomes investigation, eleven reference proteomes were uploaded. The protein sequence of the selected candidates was extracted from these eleven proteomes in a process that was followed by running multiple sequence alignment, so that, the conservation of the epitopes to be selected later could be confirmed.

### Predicting B Cell Epitopes

B cell epitopes have been predicted by bepipred linear epitope prediction method (30) which depends on a hidden Markov model approach in addition to a propensity scale method. The allergenicity and toxicity for the selected epitopes were predicted using AlgPred 2.0 and ToxinPred webservers respectively.

### Prediction of T Cell Epitopes

This stage started with the submission of filtered protein candidates to the Immune Epitope Database (IEBD) (31). MHC-I binding was predicted *via* NetMHCpan EL 4.0 prediction tool, the server recommended prediction method, and the binding prediction was performed against the reference set of HLA alleles as they represent the common binding specificities in addition to having population coverage of more than 97% (32). Regarding MHC-II binding prediction, IEBD gave a recommendation of 2.22 prediction method and again the binding prediction was run against the full HLA reference set that had population coverage of more than 99% (33). Then, the antigenic epitopes that acquired the best scores were analyzed to predict their ability to induct interferon-gamma *via* INF prediction server (34). In addition to that, epitopes’ IL-4 induction ability was predicted through the webserver (https://webs.iiitd.edu.in/raghava/il4pred/), and IL-10 induction ability was estimated through the webserver (https://webs.iiitd.edu.in/raghava/il10pred/). Finally, the allergenicity and toxicity for the selected epitopes were predicted using AlgPred 2.0 and ToxinPred webservers respectively.

### Molecular Docking of MHCI and MHCII Filtered Mono Epitopes

The final stage of epitopes prioritization before reaching the step of multitope vaccine construction was the analysis of conservancy through multiple sequence alignment and a second stage of binding assessment through molecular docking analysis. In order to run the docking study for the single epitopes, the epitope 3D structure was predicted using PEP FOLD 3 webserver (35). For MHC-II and MHC-I epitopes, crystal structures of HLA-DRB1*04:01 (PDB ID 5JLZ) and HLA-A*11:01 (PDB ID 6JP3) were selected respectively, as a molecular docking receptor. AutoDock Vina was used for Docking analysis (36).

### Construction of Multitope Vaccine

Constructing the multitope vaccine was performed by merging four components. Firstly, the β-defensin adjuvant was introduced, after that, the previously selected epitopes were incorporated and linked with suitable amino acids linkers. Finally, the inclusion of PADRE sequence, that could improve the immune response for our designed vaccine (37) was performed. This final construct was assessed for several characteristics such as antigenicity *via* VaxiJen v 2.0 (29), allergenicity using AlgPred (38), toxicity through the ToxinPred server (39), and assessed for its human homology through BLASTp against human proteome.

### Predicting Protein Solubility, Physicochemical Characters, and Secondary Structure

SOLpro server was used to predict the protein solubility upon overexpression in *Escherichia coli* (40). Several physicochemical characteristics of the vaccine were predicted including atomic composition, molecular weight, instability index, etc … using the ProtParam tool (41). Lastly, the secondary structure of the designed vaccine construct was predicted using the PSIPRED server (42).

### Prediction and Validation of Vaccine 3D Structure

We employed 3Dpro web server for the prediction of the designed vaccine tertiary structure (43). This server depends on constructing several conformations and calculates the outcome to nominate a preferred model based on energy scores. Consequently, the selected prediction would acquire the highest stability and the lowest possible energy. The next step was the usage of the GalaxyRefine server for the refinement of the selected 3D model (44). To achieve this refinement process, the side chains were rebuilt and repacked succeeded by structure relaxation *via* molecular dynamics simulation. Finally, Ramachandran plot analysis (45) and ProSA (46) were employed to validate the predicted 3D model before and after the refinement process.

### Docking Analysis between 3D Structure of Predicted Vaccine and Two Selected Targets (TLR-8 and TLR-4)

It is proposed that after multitope vaccine inoculation, it attaches to dendritic cells and macrophages through toll-like receptors (TLRs). Following that, the vaccine’s epitopes are digested by antigen-presenting cells (APC) and presented to T cells (25). Hence, TLR-8 (PDB ID: 6KYA) and TLR-4 (PDB ID: 4G8A) was brought from the protein data bank and submitted to the ClusPro 2.0 server (47) with the refined 3D structure of our vaccine, which represented the ligand, to perform a molecular docking analysis. Identifying the canonical epitope-TLR binding interactions were performed through a protein-protein interface and structural analysis using the PDBsum free web-based server (https://bio.tools/pdbsum_generate/) (48).

### Normal-State Analyses *via* Torsional Coordinate-Association

For gaining more insights regarding the collective flexibilities/motion functions of the constructed multiepitope vaccine relative to its bounded TRLs, the iMODS on-line server was used (http://www.imods.chaconlab.org/) (49). This employed server is fast and accurate while being capable of assessing the collective protein complex (epitope-TLRs) motions based on normal-state analyses of their respective internal dihedral angle (torsional) coordinates (50). Furthermore, it can predict several parameters reflecting structural flexibility/deformation reflecting significant deviation from the normal distribution values obtained from thousands deposited reference sets. Within the PDB file of both epitope-TLR complexes, the atoms/residues were continuously indexed where the number ranges 1-5824 and 5825-11649 were assigned for the TLR-4 protomer A and B, respectively, (23-627 amino acids for each protomer), while as residue ranges 1-7459 and 7460-14919 were for TLR-8 protomers (32-818 amino acids for each protomer). Subsequent residue ranges 11650-14868 and 14919-18157 were assigned for the bounded multiepitope vaccine (successive 1-349 amino acids) at the TLR-4 and TLR-8 complexes, respectively.

### Molecular Dynamics Simulation

Molecular dynamics simulation was perused for evaluating the relative epitope-TLR motions, conformation-time evolution, and thermodynamic stability under near-physiological conditions (51). Using GROMACS-5.1.4 and CHARMM36m forcefield, the constructed models of the multiepitope vaccine in complex with either TLR-4 or TLR-8 were individually simulated through 100 ns-all atom molecular dynamics as previously reported (24, 52). Protein complexes were solvated within TIP3P water and under periodic boundary conditions keeping the protein complex at 10 Å marginal distances from the box margins. Protein residues ionization states were assigned at physiological pH 7.4, while as the net charge of the entire constructed models was neutralized *via* sufficient chloride/potassium ions. Models were minimized for 0.05 ns under steepest-descent algorithm (53), and then equilibrated through two successive stages; initial NVT ensemble (Berendsen-temp couplings at 303.15 K for 1 ns) and then NPT ensemble (Parrinello-Rahmann barostat at 303.15 K and one atmospheric pressure for 1 ns). Production of molecular dynamics runs was proceeded for 100 ns under NPT ensemble and Particle-Mesh-Ewald algorithms for computing long-range electrostatic interaction, while as, LINCS was used for modeling covalent bond lengths. Both Coulomb’s and van der Waals’ non-bounded interactions were subjected to 10 Å truncations using Verlet cut-off model (54). Analysis of protein complexes were performed through estimating root-mean square deviations (RMSDs), RMS-fluctuations (RMSFs) relying on the obtained trajectory file analyses. The Molecular Mechanics/Poisson-Boltzmann (MM-PBSA) calculations, were used for estimating the free-binding energies of the simulated epitope-TLR complexes, in addition to, investigating the energy contributions of each constituting residues (55). Visual Molecular Dynamics (VMD V.1.9.3) software (University of Illinois, Urbana-Champaign, USA) was used for hydrogen bond analysis between multiepitope vaccine/TLR over the entire simulation periods. Cut-off values for hydrogen bond distance and angle were set at 3.0 Å and 20°, respectively. Conformational analysis and visualizing the simulated epitope-TLR complexes, within specified timeframes, were performed by PyMol (Schrödinger; V.2.0.6) software.

### Immune Simulation of the Chimeric Peptide Vaccine

We employed C-ImmSim server to predict the stimulated immune response after the proposed vaccine injection. We visualized the estimated immune response after the administration of three multitope vaccine injections in four weeks intervals. The followed technique represents a prime-booster-booster approach to achieve a long-lasting immune response.

### Reverse Translation and Codon Adaptation

Finally, we reached the step of codon adaptation analysis for the designed vaccine. It is an essential step to validate the sequence of the vaccine construct to be expressed in *E. coli* k-12 (the planned expression host in the future wet-lab experiments). For this purpose, JCAT server (56) was employed where the value of the codon adaptation index (CAI) calculated by the server would give a prediction for the suitability of the proposed vaccine sequence to be expressed in the selected host.

## Results

### Screening Proteins for Nominating Vaccine Candidates

By applying the antigenicity score cutoff on the MARV whole proteome, three proteins were found to have an antigenicity score that exceeded 0.5. These proteins are envelope glycoprotein with an antigenicity score of 0.54, VP30 with an antigenicity score of 0.56 and VP24 with an antigenicity score of 0.55. This was accompanied by studying the function of these three candidates in order to identify those that had physiological roles or significant virulence, proteins VP30 and VP 24 played a role in the viral replication while envelope glycoprotein has a major function of attachment to its specific host cell receptors. As a result, these three proteins were selected as final targets of epitope prediction.

### Predicting B Cell Epitopes

The B cell epitopes were predicted through IEBD webserver (**Supplementary Figure 1**) using a threshold value of 0.35. There were 10, 14, and 28 predicted epitopes for VP24, VP30, and envelope glycoprotein respectively. Then, the epitopes that had a size between 8 to 18 peptides were selected which downsized our epitopes list (**Table 1**), After that, we planned to identify the top 6 epitopes of these 3 proteins (2 epitopes for each protein) based on the antigenicity score and conservancy analysis in order to incorporate them to the multitope vaccine construct. For VP24 protein, we found only one predicted B cell epitope sized between 8:18 and this epitope had an antigenicity score less than 0.4. As a result, we modified our plan and selected 3 B cell epitopes from the other proteins (envelope glycoprotein and VP30). It is worth mentioning that all B cell filtered epitopes were predicted to be non-allergenic ad non-toxic.

**Table 1:** Predicted B cell epitopes from VP30 and envelope glycoprotein.

VP30			Envelope glycoprotein		
Epitope	Start-End	Antigenicity Score	Epitope	Start-End	Antigenicity Score
MQQPRGRSRNRS	1-12	1.11	ASNSQPQDVDSV	25-36	0.85
NLSKPPPPPKDMC	66-78	0.42	QKVADSPLEAS	57-67	0.63
PCTDPACNRDHDLD	86-99	0.54	TGVPPKNVEYTEGEEAK	74-90	1.15
NLPQDQNGVI	205-214	-0.47	PSNIRDYPKC	110-119	0.92
			KYWTSSNETQRNDT	196-209	0.025
			VTDPSGKSLLLD	97-108	-0.029
			SGSGSGEQGPHT	269-280	0.48
			EQKQSSTIL	287-295	0.66
			DKIRKDEQKEETGWGL	625-640	0.17

### Predicting T Cell Epitopes

The prediction of MHC-I epitopes resulted in 13203, 14715, and 36315 epitopes for VP24, VP30, and envelope glycoprotein respectively with a percentile rank ranging from 0.01 to 100. We selected the epitopes that showed a small percentile rank (as this small percentile rank indicates good binding properties for the epitopes) and a large antigenicity score. **Table 2** shows the top five epitopes identified for each protein. Meanwhile, the prediction of MHC-II epitopes resulted in 6453, 7209, and 18009 predictions for VP24, VP30, and envelope glycoprotein respectively. Again top-ranking epitopes are the ones that demonstrated a small percentile rank and a large antigenicity score. These top-ranked epitopes were assessed for their capability to induce INF-γ, IL-4, and IL-10. **Table 3** shows the top five epitopes identified for each protein.

**Table 2:** Top-ranked T-cell epitopes (MHC-I peptides) of VP24, VP30, and envelope glycoprotein.

No	Protein	Epitope	Antigenicity score	Allergenicity	Toxicity
1	VP24	KPSSIEIKL	1.77	Non-allergenic	Non-toxic
2	VP24	TVKWGNFIF	1.6	Non-allergenic	Non-toxic
3	VP24	IITRVNMGF	1.64	Non-allergenic	Non-toxic
4	VP24	NITEKSINL	1.62	Non-allergenic	Non-toxic
5	VP24	HISPNLLGI	1.52	Non-allergenic	Non-toxic
6	VP30	SSISVQASY	1.27	Non-allergenic	Non-toxic
7	VP30	QLPSKPQYI	0.86	Non-allergenic	Non-toxic
8	VP30	SVQASYDHF	0.82	Non-allergenic	Non-toxic
9	VP30	ENQLPSKPQY	0.67	Non-allergenic	Non-toxic
10	VP30	RSHQVALSTY	0.54	Non-allergenic	Non-toxic
11	Envelope glycoprotein	HTPPNISLTF	1.7	Non-allergenic	Non-toxic
12	Envelope glycoprotein	RTFSLINRH	1.16	Non-allergenic	Non-toxic
13	Envelope glycoprotein	EQHTPPNISL	1.13	Non-allergenic	Non-toxic
14	Envelope glycoprotein	GCFGILQEY	1.11	Non-allergenic	Non-toxic
15	Envelope glycoprotein	TTRPPIYFR	1.1	Non-allergenic	Non-toxic

**Table 3:** Top-ranked T-cell epitopes (MHC-II peptides) of VP24, VP30, and envelope glycoprotein.

No	Protein	Epitope	Antigenicity	IFN-γ inducer	IL4 inducer	IL10 inducer	Antigenecity	Toxicity
1	VP24	EWLLLEVTSAIHISP	1.28	Yes	Yes	Yes	Non-allergenic	Non-toxic
2	VP24	PFLALRILLGVALKD	1.13	Yes	No	No	Non-allergenic	Non-toxic
3	VP24	FLALRILLGVALKDQ	1.12	Yes	No	No	Non-allergenic	Non-toxic
4	VP24	SEWLLLEVTSAIHIS	0.93	Yes	Yes	Yes	Non-allergenic	Non-toxic
5	VP24	EPFLALRILLGVALK	0.91	Yes	No	No	Non-allergenic	Non-toxic
6	VP30	TSLRAALSLTCAGIR	1.09	Yes	No	No	Non-allergenic	Non-toxic
7	VP30	TNRELLLLMARKMLP	0.7	Yes	Yes	No	Non-allergenic	Non-toxic
8	VP30	TCAGIRKTNRSLINT	0.89	Yes	No	No	Non-allergenic	Non-toxic
9	VP30	CAGIRKTNRSLINTM	0.74	Yes	No	No	Non-allergenic	Non-toxic
10	VP30	LDNLTNRELLLLMAR	0.73	Yes	Yes	Yes	Non-allergenic	Non-toxic
11	Envelope glycoprotein	TRPPIYFRKKRSIFW	1.4	Yes	No	Yes	Non-allergenic	Non-toxic
12	Envelope glycoprotein	TIYFLISLILIQSIK	0.59	No	Yes	Yes	Non-allergenic	Non-toxic
13	Envelope glycoprotein	KRSIFWKEGDIFPFL	0.62	Yes	Yes	No	Non-allergenic	Non-toxic
14	Envelope glycoprotein	IYFRKKRSIFWKEGD	1.02	Yes	Yes	No	Non-allergenic	Non-toxic
15	Envelope glycoprotein	PPIYFRKKRSIFWKE	1.22	Yes	Yes	No	Non-allergenic	Non-toxic

### Molecular Docking for T Cell Epitopes

**Figure 2:**
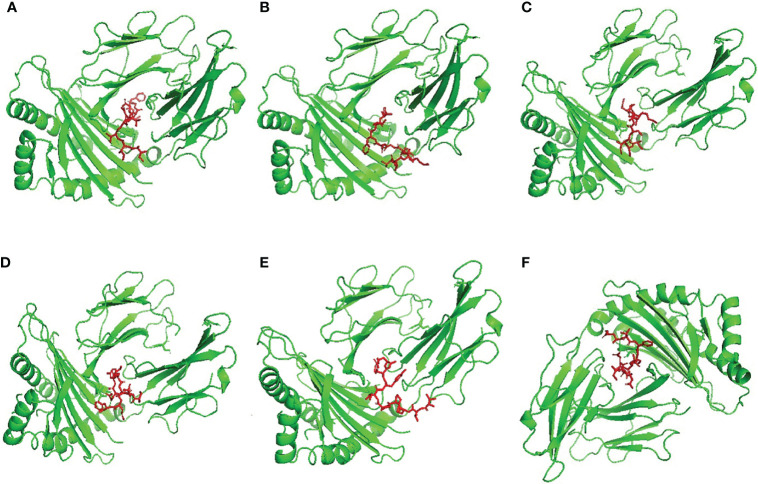
Structural positions of MHC-I epitopes (red color) in 3-dimensional structure of HLA-A*11:01 (green color), structures **(A–F)** are for epitopes number 1,2,3,4,5 and 6 respectively from **Table 4**.

**Figure 3:**
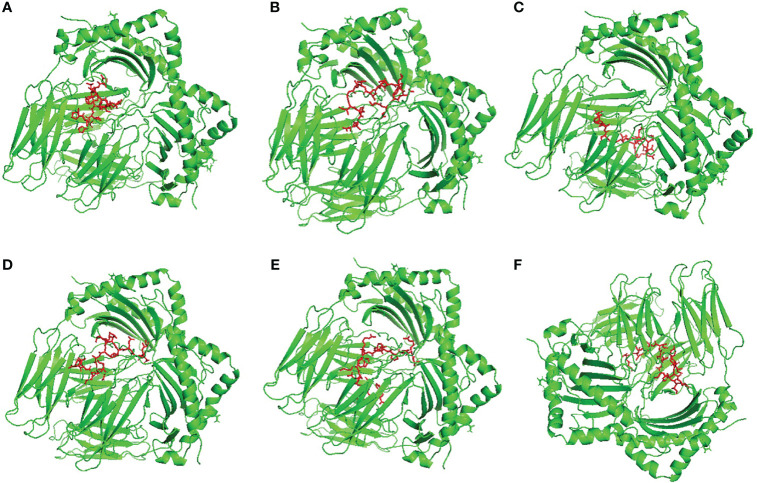
Structural positions of MHC-II epitopes (red color) in 3-dimensional structure of HLA-DRB1*04:01 (green color), structures **(A–F)** are for epitopes number 1,2,3,4,5 and 6 respectively from **Table 4**.

We selected HLA-A*11:01 and HLA-DRB1*04:01 as representative alleles to analyze the binding affinity of the filtered MHC-I and MHC-II epitopes respectively. **Figures 2**, **3** demonstrate the docked complexes of MHC-I and MHC-II epitopes respectively while **Table 4** displays the binding energy of these complexes. The binding energy scores for both types of peptides ranged between -7.2 and -9.1 (**Table 4**), and to validate these scores we investigated each of the mentioned receptors which were deposited in the protein databank with an attached ligand. we employed these ligands to act as control by removing and re-docking to their respective receptor using the same conditions of predicted epitopes docking. The docking score for these controls were -6.3 and -7.7 for HLA-A*11:01 and HLA-DRB1*04:01, respectively (the docking scores of filtered peptides were more negative than the control; therefore, they were estimated to be good binders).

**Table 4:** The binding energy of T cell epitopes with their respective allele.

No.	Epitope	MHC-I Allele	Binding Energy (kcal/mol)	Epitope	MHC-II Allele	Binding Energy (kcal/mol)
1	IITRVNMGF		-8.4	EWLLLEVTSAIHISP		-7.5
2	TVKWGNFIF		-8.4	SEWLLLEVTSAIHIS		-7.4
3	SSISVQASY	HLA-A*11:01	-8.1	LDNLTNRELLLLMAR	HLA-DRB1*04:01	-7.4
4	SVQASYDHF		-8.4	TNRELLLLMARKMLP		-7.5
5	TTRPPIYFR		-9.1	TRPPIYFRKKRSIFW		-8.7
6	RTFSLINRH		-8.0	TIYFLISLILIQSIK		-7.2

### Construction of Multitope Vaccine, Assessment of Physicochemical Characteristics and Prediction of Secondary Structure

After selecting the most promising B and T cell epitopes from VP24, VP30, and envelope glycoproteins, we moved to design the multitope vaccine depending on six CTL and six HTL epitopes (two epitopes for each protein) in addition to six BCL epitopes (three from VP30 and three from envelope glycoprotein), these epitopes were linked together using GGGS, GPGPG, and KK linkers respectively. In order to finalize the construct, we incorporated PADRE peptide sequence and beta-defensin adjuvant to reach the final sequence of our multitope vaccine which was composed of 349 amino acids and sequenced as the following:

“EAAAKGIINTLQKYYCRVRGGRCAVLSCLPKEEQIGKCSTRGRKCCRRKKEAAAKAKFVAAWTLKAAAGGGSIITRVNMGFGGGSTVKWGNFIFGGGSSSISVQASYGGGSSVQASYDHFGGGSRTFSLINRHGGGSTTRPPIYFRGPGPGEWLLLEVTSAIHISPGPGPGSEWLLLEVTSAIHISGPGPGTRPPIYFRKKRSIFWGPGPGTIYFLISLILIQSIKGPGPGTNRELLLLMARKMLPGPGPGLDNLTNRELLLLMARKKNLSKPPPPPKDMCKKPCTDPACNRDHDLDKKASNSQPQDVDSVKKTGVPPKNVEYTEGEEAKKKAKFVAAWTLKAAAGGGS”

This was followed by analyzing this final design for allergenicity, the SVM method that depends on amino acid composition predicted our designed vaccine to be non-allergen, Moreover, toxicity analysis predicted our designed vaccine to be non-toxic. Furthermore, the analysis of the antigenicity predicted our multitope construct to be antigenic with an antigenicity score of 0.61. The final design was found to have a SOLpro score of 0.993 indicating it to be soluble upon overexpression (a score that exceeds 0.5 indicating solubility upon overexpression) and did not significantly resemble human protein sequences when analyzed through Blastp (hence, the predicted vaccines would not elicit autoimmune reactions in the host). In addition, ProtParam online tools were used to further analyze the other physicochemical characteristics of this multitope vaccine as shown in **Table S1**. Finally, the secondary structure assessment predicted 23.2% helix, 26.1% strand, and 50.7% coil of the vaccine construct secondary structure (**Supplementary Figure 2**).

### Tertiary Structure Prediction, Validation and Refinement

Firstly, the 3D model of the proposed vaccine was predicted by 3Dpro webserver. Following that, the predicted model was validated before and after structure refinement on GlaxyRefine webserver. In order to perform this structural validation, we run a Ramachandran plot analysis and calculated the Z-score for the models. Regarding the primary structure, 88.5%, 11.1%, and 0.4% of residues were located in favored, allowed, and outlier regions, respectively with a Z-score of -3.68. Moving to the refined model, 93.3%, 6.3%, and 0.4% of residues were located in favored, allowed and outlier regions, respectively and the Z-score was -4.29. The refined model and its structural validation scores are shown in **supplementary Figure 3**.

### Molecular Docking of our Vaccine With TLR4 and TLR8

**Figure 4:**
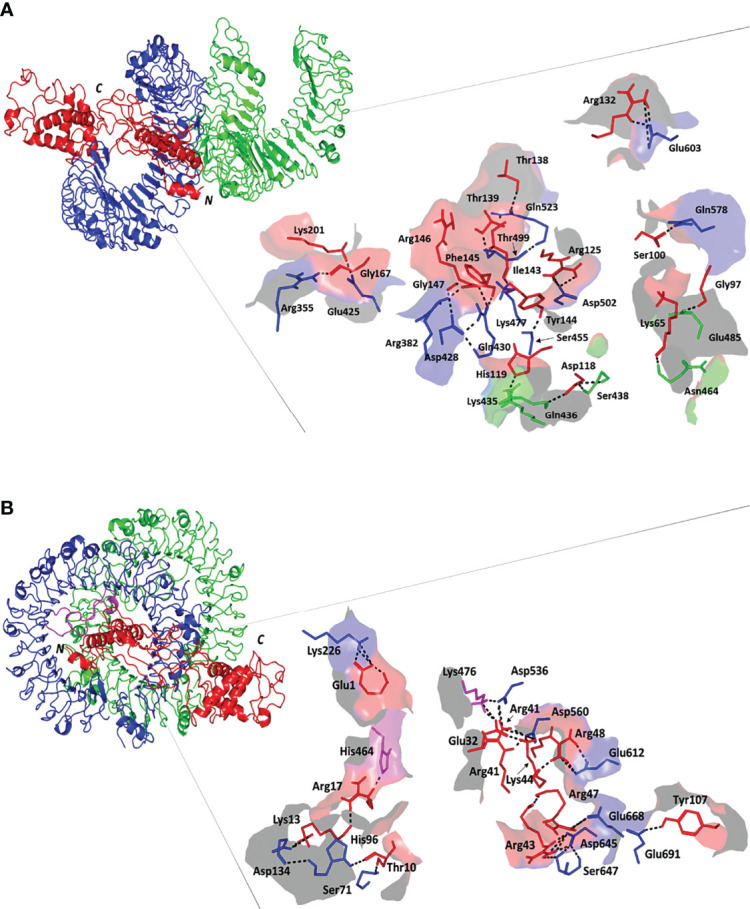
Docked complex and interface binding interactions for the designed vaccine and the two investigated TLRs; **(A)** multiepitope vaccine/TLR-4 and **(B)** multiepitope vaccine/TLR-8 complexes. 3D cartoon representation of the docked vaccine (red) at the TLR binding site (green and blue for protomer A and B, respectively). The protruding loops into the TLR-8 binding site are colored magenta. The carboxy and amine terminals of the simulated epitopes are assigned with *C* and *N*-letters, respectively. Zoomed images illustrate the surface representation of multiepitope vaccine/TLR binding polar interactions showing the residue pairs as lines, colored depending on their location within the proteins, and numbered according to their respective residue sequence. The bonds of polar interactions are shown as black dashed lines.

ClusPro 2.0 server was employed to validate the binding process between our designed potential vaccine and its relevant receptors. The predicted binding energy values were -1458 and -1386 kCal/mol for TLR-4 and TLR-8, respectively. In order to validate these values, *Brucella* Lumazine Synthase, which acts as an agonist for TLR-4 (57), was docked with the same server to TLR-4 and the smallest generated docking score was -1178.5. Based on that, the binding energy of the currently designed vaccine on TLR-4 was smaller than that of the control; therefore, a good binding is predicted for the multitope vaccine. For both investigated dimeric TLRs, the docked ligand epitope exhibited preferential binding to one TLR monomer unit over the other. The overall conformation of the epitope/TLR-4 complex showed a transverse orientation of its multiepitope vaccine across the binding cavity of TLR-4 protomer B (**Figure 4A**). Notably, the backside *N*-terminal of the docked vaccine depicted contacts with the target’s surface interface of the other monomeric unit (TLR-4 protomer A). The latter depicted 1773 Å^2^ and 774 Å^2^ interface area for the epitope in regard to 1709 Å^2^ and 748 Å^2^ for TLR-4 protomer B and protomer A, respectively. Concerning the epitope/TLR-8 docked complex, a differential ligand orientation was depicted in relation to the binding pocket of the TLR-8 target. The *N*-terminal of the docked epitope showed relevant insertion into the TRL-8 protomer B binding pocket with depicted proximity towards two target’s loops (260-268 and 433-482 residue ranges) protruding inside the binding cavity (**Figure 4B**). The inserted epitope’s section reached the dimerization interface at other TLR-8 monomeric unit (protomer A) since the latter is at a slightly drifted parallel orientation with TLR-8 protomer B. The rest of the epitope structure including its middle and carboxy-terminal rested at the surface of the target’s protomer B. Such ligand’s conformation/orientation correlated to lower interface surface areas (1677:1478 Å^2^ for epitope: protomer B and 256:234 Å^2^ for epitope: protomer A) as compared to the TLR-4 system. However, the respective TLR-8 protomer B and A interface areas were higher for the earlier protomer within a similar differential fashion to those depicted for the epitope/TLR-4 model.

Evaluation of the nature of epitope/TLR binding interactions showed interesting findings. Higher polar interaction patterns were depicted with the epitope/TLR-4 interface as compared to the TLR-8 complex. A total of three salt bridges and 21 hydrogen bond pairs were assigned for the docked epitope and TLR-4 binding site (**Figure 4A**). Depicted salt bridges were confined with TLR-4 protomer B between the residue pairs; Arg125-Asp502, Arg132-Glu603, and Lys201-Glu425 for the docked epitope and target site, respectively. Corresponding to the above-described protein-protein interface areas, higher hydrogen bonds, and other non-bonded contacts were assigned for protomer B (15 and 144) in regard to protomer A (15 and 144 versus 6 and 73, respectively). Moving towards the epitope/TLR-8 predicted complex, lower extent of binding interactions were illustrated through the protein-protein interface analysis. Nine salt bridges and 17 hydrogen bonds were depicted between epitope and TLR-8 protomer B, yet no polar interaction was illustrated at the epitope/protomer A interface (**Figure 4B**). Salt bridges were between the residue pairs; Glu1-Lys226, Lys13-Asp134, Glu32-Lys476, Arg41-Asp536, Arg41-Asp560, Arg43-Asp645, Lys44-Asp560, Arg47-Glu668, and Arg48-Glu612 for epitope/TLR-8 protomer B, respectively. On the other hand, limited non-bounded contacts (135 and 7) were illustrated at the interface between docked epitope and TLR-8 protomer B and A, respectively. The detailed atom-atom interactions and bonding distances, as well as representative diagrams for the depicted epitope/TLR protomer interfaces, are thoroughly described in **Tables S2, S3**.

### Normal-State Analyses *via* iMODS Server

**Figure 5:**
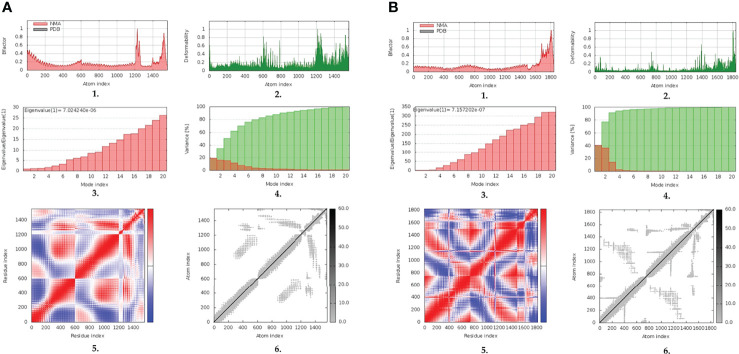
Normal-state analyses *via* iMODS server for the docked multitope vaccine/TLR complexes; **(A)** multitope vaccine/TLR-4; **(B)** multitope vaccine/TLR-8 models. Ligand-receptor interaction was assessed throughout comparative **1.** B-factor indices, **2.** deformability, **3.** variances, **4.** eigenvalues, **5.** covariance of residue indices, and **6.** elastic network analyses.

The inherited stability and conformational mobility of both the docked vaccine and TLRs were analyzed based on the torsion angle-related normal state analysis using the iMODS server. Interestingly, the estimated B-factors were significantly higher for the docked vaccine at both TLR models with higher values being associated with the ligand’s *C*-terminal residues (**Figures 5A1, B1**). On the other hand, the target TLR-4 protomers showed higher inherited flexibility in relation to those of the TLR-8 target. Generally, the B-factor correlates to the relative magnitude of atom displacement around conformational equilibria. Findings of B-factor analyses were recapitulated by the obtained complex deformability index presented in **Figures 5A2, B2** where higher individual distortions were assigned for vaccine amino acids, particularly at terminal residues, as compared to those of the bound TLR target proteins. The estimated eigenvalues representing the motion stiffness of each vaccine/TLR complex were 7.02 x 10^-06^ and 7.16 x 10^-06^, for TLR-4 and TLR-8 complexes, respectively. These values are inversely proportional to variance predicting the significantly higher mobility of the vaccine as compared to the TLR ones across collective functional motions (**Figures 5A3,4, B3,4**). The server provided the covariance matrix illustrating the coupled residue pairs demonstrating uncorrelated, correlated, or anti-correlated motions as white, red, and blue colors, respectively. Both docked vaccines had less anti-correlated motions as well as higher correlated residue-pair motions than those of the bound TLRs (**Figures 5A5, B5**). Finally, the obtained elastic-network model explains the differential flexibility patterns among both the vaccine and bound TLR (**Figures 5A6, B6**). The elastic-network model illustrates the atom pairs linked *via* springs based on stiffness degree between them relying on different color representations. Typically, stiffer strings correlate to dark gray colors. Along the normal distribution of stiffer string, the docked vaccines showed discontinuous dark-gray bands where it was of more discontinuous strings within the TLR-8 model. On the contrary, the target TLR residues more continuous gray bands around the same immobility normal string.

### Analysis of Molecular Dynamics Simulation Runs

**Figure 6:**
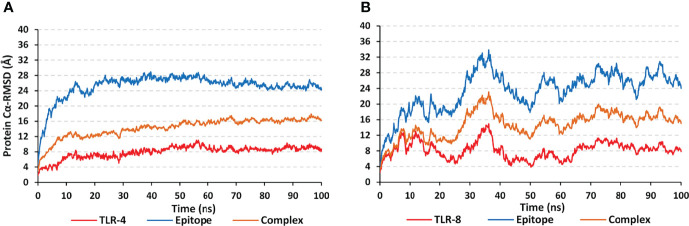
Trajectory-based thermodynamic stability analysis of the simulated multiepitope vaccine in complex with different TLRs along 100 ns all-atom simulation runs. The estimated *Cα* RMSDs (Å) of simulated **(A)** epitope/TLR-4 model; **(B)** epitope/TLR-8 model, were represented as a function of molecular dynamics timelines (ns).

The simulated multiepitope vaccine models showed differential thermodynamic stability profiles in regard to the kind of the bounded TLR target. The alpha-carbon RMSD trajectories (*Cα* RMSDs) for the simulated epitope/TLR-4 model showed typical dynamic behaviors (**Figure 6A**). Throughout the initial simulation frames, the *Cα* RMSD values increased gradually owing to the release of the previously applied constraints at the minimization and equilibration stages. Relaxation of the system model was proceeded till around the first 20 ns where afterward the protein *Cα* RMSDs attained respective equilibration plateau till the end of the runs (100 ns). Rapid equilibration and steady *Cα* RMSDs were maintained for more than half the simulation runs (> 70 ns) regarding the epitope, TLR-4, and their respective combined complex. Across each respective equilibration plateau, the epitope’s average *Cα* RMSD values were higher than those of the corresponding bound TLR-4 protein target (24.27 ± 1.11 Å versus 8.73 ± 0.71 Å). Regarding the other simulated model, epitope/TLR-8, significant *Cα* RMSD fluctuations were depicted for both the simulated epitope and bound target (**Figure 6B**). Fluctuations were much more profound around the 30-40 ns and within initial simulation timelines. Nevertheless, near equilibration plateaus were attained following the late simulation times (beyond 65 ns) showing limited fluctuations. Notably, much higher *Cα* RMSD fluctuations were depicted for the ligand epitope as compared to its bound TLR-8 target (average 26.24 ± 2.09 Å versus 8.71 ± 1.42 Å) following respective thermodynamic equilibrations. Interestingly, both the ligand and target proteins were converged down to *Cα* RMSD values being comparable to their corresponding protein within the other epitope/TLR model near the end of the molecular dynamics simulation (100 ns).

**Figure 7:**
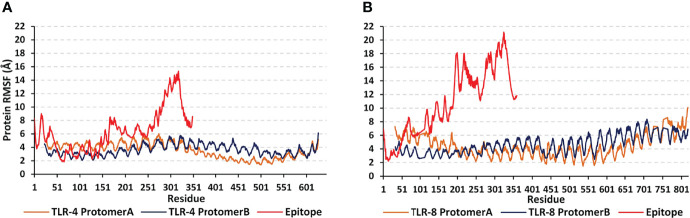
Global stability analysis and residue-wise mobility trends of the simulated multiepitope vaccine in complex with different TLRs along 100 ns all-atom simulation runs. The protein’s *Cα* RMSFs (Å) of simulated **(A)** epitope/TLR-4 model; **(B)** epitope/TLR-8 model, were represented as a function of constituting residue sequence numbering; TLR-4 protomer A = 27-627; TLR-4 protomer B = 23-627; TLR-8 protomer A = 32-818; TLR-8 protomer B = 33-818; multiepitope vaccine = 1-350 amino acid numbering.

For gaining more insights regarding the simulated protein global stability and residue-wise flexibility contributions, the *Cα* RMSF trajectories were monitored across the entire 100 ns simulation runs (**Figure 7**). Simulated proteins of both systems showed typical dynamic behaviors where terminal residues, as well as their vicinal amino acids, depicted higher motion patterns (elevated *Cα* RMSFs) in regard to their central core ones. The latter *Cα* RMSF fluctuations were of higher values for the simulated epitopes in regards to their bounded TLR protein targets. Interestingly, both TLR-4 protomers were assigned with comparatively lower mobility/fluctuation profiles as compared to those of TLR-8 ones (3.62 ± 0.80 Å versus 4.64 ± 1.32 Å). Similarly, higher stability/immobility patterns were also depicted for the TLR-4 bounded epitope in relation to that of the TLR-8 model (6.44 ± 3.10 Å versus 10.91 ± 4.94 Å). It is worth mentioning that the simulated epitopes of both models showed the most recognized flexibility/mobility trends for their respective *C*-terminal residues as well as vicinal amino acids (high numbered amino acid sequencing).

**Figure 8:**
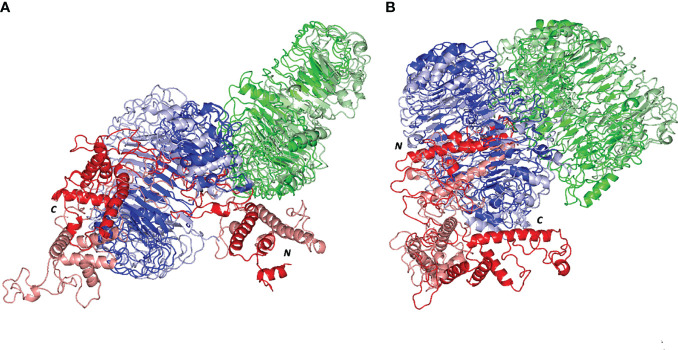
Comparative dynamic conformation/orientation for the simulated multiepitope vaccine/TLR models. Aligned initial and final simulated trajectories of each model; **(A)** multiepitope vaccine/TLR-4; **(B)** multiepitope vaccine/TLR-8. Proteins are represented as 3D cartoons and differently colored in green, blue, or red for respective TLR protomer-A, TLR protomer-B, or the epitope proteins, as well as, in dark or light colors in regard to their respective extracted 0 ns or 100 ns trajectories. The carboxy and amine terminals of the simulated multiepitope vaccines are assigned with *C* and *N*-letters, respectively.

Subsequent conformational analysis was proceeded to grasp the main time-evolution conformational changes within the different epitope/TLR models. This was performed through comparative conformational analysis of simulated models at the initial and final dynamic trajectories (58). Extracted frames (0 ns and 100 ns) were subsequently minimized down to 1x10^-3^ Kcal/mol.A^2^ gradient *via* MOE2019.01 package before being visually analyzed *via* PyMol software. Interestingly, the simulated epitope managed to be confined within the binding sites of both investigated TRLs, while exhibiting stable binding complex states (**Figure 8**). More significant conformational alterations were depicted with the simulated epitopes in comparison to their respective bound TLR targets. The latter epitope alterations allowed these ligands to adopt more compacted conformations with orientations being directed towards the TLR lateral interfaces and binding sites. It is worth noting that the higher comparative dynamic alterations were illustrated for the *C*-terminal region of the simulated epitopes as compared to their amine ends. Regarding both simulated epitope/TLR-4 versus TLR-8 models, the earlier depicted less profound dynamic conformational alteration across the simulated runs. This was highly observed within the multiepitope vaccine/TLR-4 model where lower aligned *Cα* RMSD values between respective 0 ns and 100 ns trajectories were depicted (5.590 Å and 9.923 Å, for multiepitope vaccine/TLR-4 and TLR-8 models, respectively).

Evaluation of the total ligand/TLR free-binding energies (Δ*G_total_*) showed significant affinity of the designed vaccine with higher preferentiality (higher negative values) towards TLR-4 as well as its protomer B in regard to TLR-8 and cognate protein, respectively (**Table 5**). For identifying the nature of vaccine/TLR binding, the obtained total free-binding energy was dissected into its constituting energy terms in regards to the hydrophobic van der Waal potentials (Δ*G_van der Waal_*), Coulomb’s electrostatic energies (Δ*G_electrostatic_*), polar solvation (Δ*G_solvation_*), and non-polar solvation (Δ*G_SASA_*) energy contributions. Notably, the Δ*G_electrostatic_* within both vaccine/TLR models depicted superior energy contribution for the Δ*G_electrostatic_* interactions over the hydrophobic potentials reaching up to several folds. Within both simulated models, minimal polar solvation energy was assigned for protomer A in regard to protomer B, being of particular differential values for the vaccine in bound with TLR-8 target. On the contrarily, higher apolar solvation energy term contributions were assigned for the TLR’s protomer B over those of protomer A, showing the greatest value for the TRL-4 complex system.

**Table 5:** Free energies of binding and their individual energy contribution terms for multiepitope vaccine/TLR complexes.

	Multiepitope vaccine/TLR-4 complex				Multiepitope vaccine/TLR-8 complex			
Energy(kJ/mol ± S.D.)	Protomer A	Protomer B	Combined		Protomer A	Protomer B	Combined	
**Δ*G_van der Waal_***	-315.619 ± 51.163	-629.274 ± 41.117	-944.893 ± 56.14		-114.891 ± 21.397	-390.993 ± 50.268	-505.884 ± 45.833
**Δ*G_electrostatic_***	-5732.477 ± 164.269	-9604.370 ± 101.274	-15336.847 ± 132.7715		-2576.698 ± 79.934	-7126.549 ± 147.729	-9703.247 ± 43.833
**Δ*Gs_olvation_***	572.009 ± 98.711	1577.708 ± 112.365	2149.717 ± 105.538		17.459 ± 140.784	2216.553 ± 163.088	2234.012 ± 101.936
**Δ*G_SASA_***	-37.303 ± 5.591	-83.648 ± 1.513	-120.951 ± 3.552		-13.651 ± 5.475	-69.720 ± 13.213	-83.371 ± 11.204
**Δ*G_total_***	-5513.390 ± 89.208	-8739.584 ± 61.622	-14252.974 ± 77.325		-2687.781 ± 103.224	-5370.709 ± 75.238	-8058.490 ± 81.431

**Figure 9:**
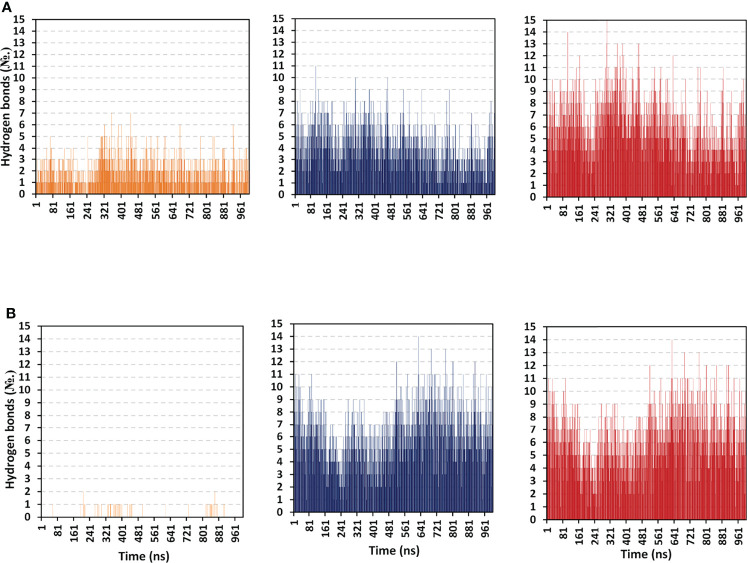
Hydrogen bond number-time evolution within the simulated multiepitope vaccine/TLR models. **(A)** multiepitope vaccine/TLR-4; **(B)** multiepitope vaccine/TLR-8 models. Left, middle and right panels are respective for the vaccine/TLR protomer A, protomer B, and whole target protein complexes.

Further exploration of the differential Δ*G_electrostatic_* protein-protein binding interactions between both TLR models as well as cognate protomers, hydrogen bond analysis was conducted across the simulated trajectories (**Figure 9**). As a general observation, vaccine binding towards the target’s protomer B illustrated the greater range of h and TLR-8 bound complexes (average hydrogen bond №; ~ 4 ± 1.87 and 6 ± 2.19, respectively). On the contrarily, a moderate to a limited number of the hydrophilic hydrogen bonds were depicted for the TLR-4 protomer A (~ 2 ± 1.27 H-bonds) as well as its corresponding protein at TLR-8 system (< 1 ± 0.25 H-bonds). Interestingly, patterns of hydrogen bonding at the vaccine/TLR-4 complex were higher at the first half of the simulation runs as compared to the late simulation timeframes. These polar protein-protein binding patterns were inversed for the TLR-8 bounded complex where higher numbers of hydrogen bonding were depicted beyond the protein system convergence and across the respective equilibration plateau (> 70 ns timeframes).

Across the 100 ns simulated run of vaccine/TLR-4 system, significant hydrogen bond frequencies (% occurrence of 100 ns time) were assigned for the vaccine-TLR residue pairs; Gly21-Arg496 (29.11%), Arg22-Asp490 (49.01%), Cys23-Ser518 (38.12%), Lys88-Glu608 (46.14%), Gly97-Glu485 (28.22%), Tyr107-Glu605 (24.75%), Tyr114-Asp502 (35.46%), Ser116-Gln484 (26.24%), Arg125-Asp550 (60.89%), Asn131-Glu603 (27.23%), Arg132-Glu603 (55.45%), and Arg140-His426 (60.89%). Concerning the inbound TLR-8 model, the following vaccine-TLR residue’s hydrogen bond pairs were correlated to significant occupancies; Glu1-Glu129 (26.63%), Glu1-Glu150 (22.57%), Lys5-Glu133 (47.52%), Arg19-Asp462 (40.69%), Glu32-Lys476 (33.17%), Thr40-Asp645 (20.99%), Arg41-Asp536 (73.76%), Arg41-Asp560 (48.02%), Lys44-Glu612 (50.10%), Arg47-Glu668 (67.33%), Arg48-Glu612 (99.56%), Lys50-Glu768 (42.08%), and Lys88-Glu691 (64.65%). It worth noting that the residue pairs of TLR-8 model were exclusively related to the protomer B rather than protomer A target protein.

**Figure 10:**
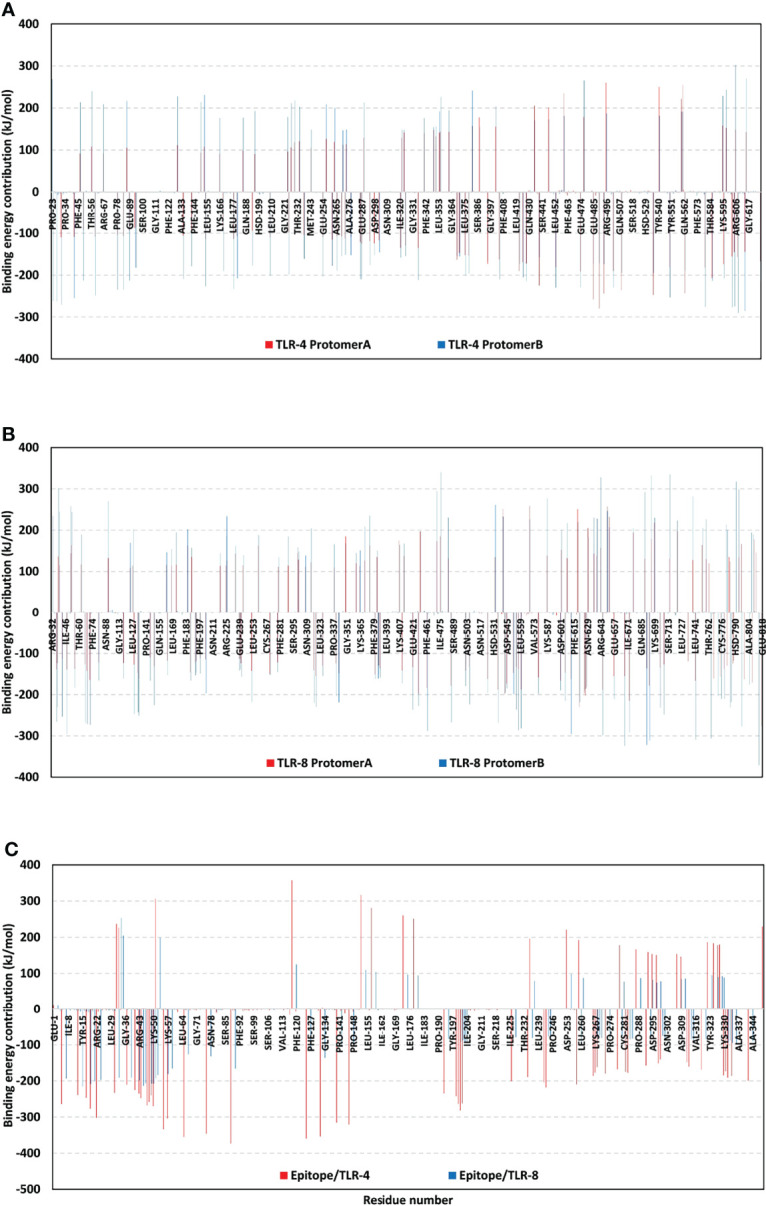
Residue-wise free binding energy contributions for simulated multiepitope vaccine/TLR models. **(A)** bounded TLR-4 protomers A and B; **(B)** bounded TLR-8 protomers A and B; **(C)** bounded multiepitope vaccine at TLR-4 and 8 binding sites. Regions of negative-valued **Δ*G*** kJ/mol confer highly favored binding forces of attraction, while as those of positive-valued **Δ*G*** kJ/mol confer unfavored binding forces of high-repulsions.

To further highlight the significance of the above-described hydrogen bond-residue pairs within the multiepitope vaccine/TLR complex stability, the Δ*G_Total binding_* was further decomposed into the protein’s residue-wise energy contributions (**Figure 10**). Findings within the latter Figure showed higher positive-values energy contributions (repulsive forces) for the TLR-8 protomers as compared to those of TLR-4 ones. Energy contributions were more distributed across the residues of both TLR-4 protomers. On the contrarily, the TLR-8 protomer A residue-wise energy contributions were almost concentrated for the *C*-terminal residues. Regarding the bounded multiepitope vaccines, higher residue-wise energy contributions were assigned for that in bound to TLR-4 and particularly towards the epitope’s *N*-terminal side. the highest negative-value energy contributions were assigned for the above-described vaccine’s residue which was identified to mediate relevant hydrogen bonding pairs. The highest energy binding residues included; Arg22 (-302.37 kJ/mol), Lys88 (-373.25 kJ/mol), Arg 125 (-359.65 kJ/mol), Arg132 (-353.73 kJ/mol), and Arg140 (-315.34 kJ/mol) for epitope/TLR-4 model. on the other hand, Lys5 (-192.88 kJ/mol), Arg19 (-200.52 kJ/mol), Arg41 (-198.69 kJ/mol), Lys44 (-206.76 kJ/mol), Arg47 (-207.69 kJ/mol), Arg48 (-207.42 kJ/mol), Lys50 (-183.53 kJ/mol), and Lys88 (-165.52 kJ/mol) were assigned of highest energy binding contributions within epitope/TLR-8 complex system.

### Immune Simulation of the Designed Vaccine

The generated immune response as a result of the proposed vaccine successive injections is shown in **Supplementary Figure 4**. Firstly, the multitope vaccine stimulated the formation of high levels of IgM + IgG, where these antibodies demonstrated a growing level trend with successive injections. Secondly, several cytokines were predicted to be stimulated following the proposed vaccine injection where INF-γ showed the highest level of induced cytokines. lastly, both B and Th cells experienced a high increase with successive vaccine doses and the highest level of both cells was obtained following the second booster dose injection.

### Vaccine Reverse Translation in Addition to Codon Optimization

Finally, we reached the last stage where the computational analysis included a reverse translation and codon optimization of our multitope vaccine using JCat server for this purpose. The analysis of the amino acid sequence of our vaccine showed GC content of 52.9% indicating an accepted value as the accepted range ranged from 30 to 70%. Moreover, calculating Codon Adaptation Index (CAI) indicated a high probability of protein expression with a value of 1, when transferring these experiments to the wet lab, the CAI value ranges from 0 to 1 with the accepted range ranges between 0.8 and 1.

## Discussion

MARV is a lethal zoonotic virus that can lead to a series of symptoms starting from abdominal or chest pain and develops into a massive hemorrhage with multi-organ dysfunction (59). Currently, there is no specific treatment for this viral hemorrhagic fever and patients receive only supportive treatment with electrolytes, pain killers, and antibiotics to inhibit the secondary infection (60). Due to its high fatality rate and successive outbreaks, the development of an effective vaccine against this deadly virus is a major health priority (61). In addition to the traditional approaches for vaccine development, the great revolution in the sequencing methods and the availability of huge data regarding pathogens’ genomes have led to a growing methodology for vaccine designing that would save both cost and time (62). The continuous development of the computational tools and servers that deal with the genomic and proteomic data have revolutionized the approach of the *in silico* vaccine prediction that in turn has been extended to involve bacteria, viruses, fungi, and even cancer (63). Examples of targeted viruses for designing an epitope-based vaccine through a computational approach includes Zika virus (64), Influenza A virus (65), African Swine Fever Virus (66), and COVID- 19 (67)

The application of immunoinformatics for predicting vaccine candidates against MARV has been applied in previous trials. A recent study (68) revealed an epitope-based vaccine based on predicted B and T cell epitopes of RNA Dependent RNA Polymerase Protein. Another study (69), targeted the same enzyme for the prediction of B and T cell epitopes and nominated the best-selected epitopes as vaccine candidates against MARV. On the other hand, unlike previous trials that targeted one protein candidate without specific rationale, we filtered the MARV whole proteome based on the antigenicity and the virulence of each protein and came out with three proteins namely VP24, VP30, and envelope glycoprotein. VP24 has a major role in the formation of viral infectious particles. It was reported that silencing of VP24 through molecular biology techniques would not affect the viral transcription or replication. Instead, the release of viral particles was significantly impaired which supports the role of VP24 in the interaction between nucleocapsid and budding site at the plasma membrane (70). Moving to VP30, it was reported that knockdown of VP30 in MARVinfected cells results in a significant reduction in the total viral proteins production in addition to the inhibition of viral particles release (71). The last candidate, envelope glycoprotein, mediates the process of MARV attachment and entry into its target receptors of infected cells (72), and for this reason, it was selected as a potential MARV vaccine target (73). The next stage after the detection of vaccine candidates is the prediction of B and T cell epitopes. Usage of epitopes instead of the whole proteins has the advantage of targeting the antigenic parts of proteins and reducing the probability of adverse allergic reactions (74). The limited immunogenicity of single-epitope peptides moves the prediction process to design a multitope vaccine instead where vaccine construct with several epitopes would have an improved antigenicity and immunogenicity especially when an appropriate adjuvant sequence is incorporated (75). This was the strategy of the current study which demonstrated an advantage over studies (68) and (69) that applied a similar approach for designing a MARV vaccine.

The current study has either applied the default thresholds suggested by the prediction severs or more strict scores for more stringent filtered results. For example, VaxiJen 2.0 server, which was utilized for antigenicity prediction, assumes that the peptides with a score of more than 0.4 are probable antigens but in the current study we put our threshold at 0.5. On the other hand, the prediction of B cell epitopes was performed based on the default threshold suggested by the prediction server. Moving to the prediction servers and applied algorithms, we applied NetMHCpan EL 4.0 prediction tool for the estimation of MHCI peptides, and this tool’s performance was assessed on two independent external data sets; one consisting of 15,965 eluted ligands covering 27 HLA molecules, and another consisting of 1,251 validated CTL epitopes covering 80 HLA molecules reported in the IEDB (76). We also used bepipred linear epitope prediction method for the estimation of B cell epitopes, a tool that when tested on the validation data set showed a significantly better performance than any of the other tested methods (30). For the antigenicity assessment, VaxiJen 2.0 was used. This server applies an alignment-free approach for antigen prediction, which is based on auto cross-covariance (ACC) transformation of protein sequences into uniform vectors of principal amino acid properties. The models performed well in validation and showed a prediction accuracy of 70% to 89% (77). In addition to the validation stage that was applied during the development of these tools, another source of validation is the protection of the epitopes, that were predicted through these tools, against several pathogens when they were assessed for their activity in wet-lab experiments (28, 78). It is important to mention that the prediction of these tools is not 100% true and here comes one of the major advantages of the multi-epitope vaccines over the ones that are based on mono epitope which is the involvement of more than one epitope that would reduce the probability of the production of a non-effective final vaccine due to a prediction error.

In the current study, the single predicted epitopes were filtered based on several criteria such as the percentile rank, the antigenicity score, the degree of binding to a representative allele, and the conservancy of these epitopes. Moreover, the prediction was initially performed against a reference list of alleles to provide a high percentage in terms of population coverage. For the multitope construction, amino acid linkers were used to link top-ranked single epitopes and assure the effective separation of the assembled mono epitopes *in vivo* (79). The first linker, EAAAK, was used to enhance the bi-functional catalytic activity, and give stiffness in addition to enhancing fusion protein stability. The second linker, GPGPG, was selected for its ability to induce HTL immune response and the ability to break the junctional immunogenicity, resulting in individual epitopes’ restoration of immunogenicity. The final linker, KK, was employed because of its ability to bring the pH value close to the physiological range (21). Moreover, the beta-defensin adjuvant and PADRE peptide were added to finalize the proposed vaccine construct with their added roles of potentiating the immune response and minimizing the HLA polymorphism in the population (80). Selected T cell single epitopes were docked in a representative receptor as a primary step to validate their reactivity (81) where the binding energy of the docked complex supported the nomination of top predicted epitopes. Following the multitope vaccine construction and before predicting its three-dimensional structure, the proposed vaccine construct was analyzed for its characteristics and was found to be soluble upon over-expression, antigenic, non-allergen, and non-toxic. Other assessed physicochemical characteristics demonstrated that the instability index was 38.9 indicating that the construct is stable, the aliphatic index was 73.3 indicating that the construct is thermostable, and the negative GRAVY score (-0.33) indicated that the construct is hydrophilic. These promising results moved our study to the next steps of tertiary structure prediction and docking analysis. The proposed vaccine tertiary structure was predicted and refined computationally and the validation scores showed a high quality of the predicted model.

The final stage of the current study was a molecular docking analysis between the designed vaccine and TLR-4 and 8 where the low binding energy scores gave a primary indication that a good binding is predicted to occur between the proposed vaccine model and its targets and to get a closer view of the docked complex behavior, we employed normal mode analysis that was integrated into the iMODS server and the output data, that described the collective functional motions of the complex, demonstrated promising stability of the complex that was deeply analyzed in a molecular dynamics stimulation study.

The thermodynamic stability was illustrated for the designed multiepitope vaccine towards two TLR targets within the conducted 100 ns all atom dynamics simulations. Both systems were successfully converged since comparable *Cα* RMSD values were depicted for each corresponding protein of opposite simulated models at the end of the simulation runs. Moreover, the differential *Cα* RMSD values between the multiepitope vaccine and its bound TLR target were within a 3-fold difference conferring successful protein convergence needing no further simulation extensions. Generally, *Cα* RMSD is the stability-indicating parameter that estimates the molecular deviations from the respective reference molecule at the initial frame. This tool has been applied to validate molecular dynamics simulation as well as ensure the significant ligand/target thermodynamic stability and confinement through furnishing low *Cα* RMSD values and achieving rapid equilibration (82, 83). The here depicted ligand/TLR *Cα* RMSD-based stability profiles were highly comparable to those obtained from several reported studies investigating computationally designed multitope vaccines targeting human TLRs (84–86).

The significant preferentiality of our designed multiepitope vaccine towards the TLR-4 was also illustrated where the latter showed earlier *Cα* RMSD equilibration and steadier *Cα*-RMSD tones levelling up for more than 70 ns of the simulated runs. This could be reasoned for the differential orientation/conformation of the simulated multiepitope vaccine within each TLR binding site. The presence of two protruding loops (260-268 and 433-482 residue rages) into the TLR-8 binding pocket could have hindered the epitope/TLR-4-like transverse orientation across the TLR-8’s inner pocket. The binding of the designed vaccine at the lateral interface of TLR-8 was also depicted through several reported studies. Three research groups showed significant affinity of a novel engineered anti-SARS-CoV-2 vaccines towards TLR-8 target (84–86). Despite the differential vaccine size at each study, both vaccines predicted a relevant anchoring at the TLR-8 target’s dimerization lateral interface. A study by Sana et al. also depicted a lateral docking orientation of anti-Crimean Congo hemorrhagic fever virus multi-valent vaccine towards three human TLRs, including the TLR-8 (86). Thus, our depicted docking findings were considered valid and highly reliable being in great condcordance with several reported data.

Validation of the adopted docking poses of the epitope/TLR-4 and -8 complex was further proceeded through investigating the RMSF tones across the molecular dynamics simulated trajectories. Typically, the RMSF-based analysis represents a residue-wise flexibility assessment tool that permits an estimation of the protein amino acids’ average deviation from their reference position. Such analytical parameter would provide information regarding the inherited flexibility/mobility of the simulated protein’s down to their own constituting amino acid levels (87). In our study, the simulated TLR proteins depicted typical fluctuation/thermodynamic mobility patterns being comparable with those observed within several previously reported *in-silico* studies (24, 85). However, the differentially higher RMSF values for the simulated multiepitope vaccines in regard to their bound TLRs were mostly related to the protein’s respective higher structural folding and/or packing. Having the simulated TLRs at their higher oligomeric states (Homo-2-mer-A2) would rather infer minimal flexibility and higher immobility (88) as compared to the simulated monomeric multiepitope vaccines. Additionally, both TLRs exhibit a highly-dense packing shoe-like ternary protein structure with several high-ordered *β*-sheets. The latter would impose lower RMSF values for TLR as compared to the multiepitope vaccines where the latter possesses extended *α*-helices with interconnecting flexible *β*-loops of long-to-medium lengths. These vaccine/TLR differential flexibility profiles were also illustrated at the higher RMSDs for the overlaid initial/final timeframes as well as at the normal-state analyses developed *via* the iMODS server. The latter approach showed more uniform stiffness trends, as well as lower mobility indices, deformability, and B-factor values for TLRs in regard to the bounded vaccines.

Both the investigated MM/PBSA-based binding-free energy calculations and hydrogen bond analysis emphasized the preferential affinity of the bound vaccine towards the TLR-4 binding site over that of TLR-8 target protein. Showing higher negative-value total energies and pronounced electrostatic energy term contributions have validated the significance and comparative patterns of the vaccine’s polar interactions with bound TLR-4/B as well as their cognate protomers A/B previously described within our preliminary docking findings. Finally, the conducted molecular dynamics simulations provided further validation of the conducted docking study based on energy contributions and bonding interactions. Both furnished residue-wise energy contributions and high-frequency hydrogen bond residue pairs were representative to the preliminary docking protein-protein interaction findings reported within this study.

Although limited polar/hydrogen bonding patterns were shown for the bound vaccine/TLR-8 protomer, the extended contacts with the other cognate (protomer B) have managed to overcompensate. This benched assistance allowed the vaccine/TLR-8 complex to exhibit an overall binding profile being comparable to that of the epitope/TLR-4 model. The latter highlights the significance of the vaccine’s *N*-terminal conformation/orientation shift towards the TLR-8 dimerization interface for stabilizing the epitope/TLR complex beyond 70 ns and till the simulation end. Again, such fundamental shift was reasonably depicted through the increased hydrogen bond profile beyond the epitope/TLR-8 protein convergence and throughout their respective dynamic equilibration plateau. It is worth noted that such conformational/orientation shift would have greatly counterbalanced the predicted electrostatic penalties and polar solvation energies (Δ*G*_Solvation_) arose during the multiepitope vaccine ligand binding at TLR-8 binding site. Generally, solvation energies confer significant forces of repulsive against the ligand-binding process since such processes rely on solvent-displacement. Despite the depicted shift, the polar solvation energy terms were shown higher within the multiepitope vaccine/TLR-8 model as compared to those of the TLR-4 one. This could confer the preferentiality of the vaccine’s transverse orientation at TLR-4 as compared to TLR-8, while as further signify the TLR-8 protruding loops that would impose great challenge against vaccine/TLR-8 proper anchoring. This was highly rationalized since several reported *in-silico* studies, including our presented data, showed a significant docking of their designed vaccines towards the TLR-8 lateral interface while depicting relevant thermodynamic stability throughout variable molecular dynamics simulation time runs (84–86).

Another interesting finding was presented at our residue-wise energy contribution analysis where the depicted solvation energies were majorly mediated *via* the TLR residues rather than those of the vaccines. This could be reasoned for the high-ordered water molecules along the hydrophobic surface of the multiepitope vaccine/TLR binding site. On the other hand, the total non-polar interactions (Δ*G*_van der Waal_ + Δ*G*_SASA_) were shown to be higher at the TLR-4, particularly for the protomer B, conferring it respective larger binding surface area as well as higher hydrophobic potentialities towards vaccine anchoring. This speculation could be rationalized since accumulated evidence has considered the investigated TLRs’ binding sites to be extended and of more hydrophobic nature (89). Based on all above evidence, it was satisfactory to say that the designed multiepitope vaccine showed significant binding affinity towards the two human TLR binding pockets at their biologically active higher oligomeric states (homo-2-mer-A2), yet with significant preferentiality towards that of TLR-4 target protein.

## Conclusions

Application of computational approaches for vaccine design before validation through wet lab techniques is a modern path that has been applied extensively in the last few years with the advantage of being a great economical solution that saves both cost and time. Proteome exploration of MARV recommended three antigenic proteins (VP24, VP30, envelope glycoprotein) with essential physiological and pathological roles as vaccine candidates. Utilization of *in silico* tools for the prediction of B and T cell epitopes then assembly of a multitope vaccine came up with a potential vaccine construct having promising physicochemical and immunological characteristics. In addition to that, validation of both mono and multiple epitopes through molecular docking-coupled dynamics simulation analysis would support the nomination of the currently designed vaccine as a putative solution against MARV. We recommend directing this vaccine to the next stage of biological assessment for validating our findings.

## Data Availability Statement

The original contributions presented in the study are included in the article/**Supplementary Material**. Further inquiries can be directed to the corresponding authors.

## Author Contributions

MAS, WKA, AMR, MA, MAE, and KMD: conceptualization, methodology, and original draft preparation. SA, EF, SMAS, SSE., and RAE: writing— review, and editing. AAB, SSE, and RMD: Funding. MAS and MAE: supervision and project administration. All authors contributed to the article and approved the submitted version

## Funding

This research was funded by the Deanship of Scientific Research (DSR) at King Abdulaziz University (KAU), Jeddah, Saudi Arabia, under grant number (RG-1-166-43).

## Conflict of Interest

The authors declare that the research was conducted in the absence of any commercial or financial relationships that could be construed as a potential conflict of interest.

## Publisher’s Note

All claims expressed in this article are solely those of the authors and do not necessarily represent those of their affiliated organizations, or those of the publisher, the editors and the reviewers. Any product that may be evaluated in this article, or claim that may be made by its manufacturer, is not guaranteed or endorsed by the publisher.
