# Supplementary material for: Mining of Marburg Virus Proteome for Designing an Epitope-Based Vaccine
Source: Front Immunol. 2022 Jul 15;13:907481. doi: 10.3389/fimmu.2022.907481 (PMC9334820; doi:10.3389/fimmu.2022.907481)
Supplement: Supplementary file 1 [file DataSheet_1.docx]

Supplementary Materials

[Mining of Marburg Virus Proteome for Designing an](https://www.tandfonline.com/doi/abs/10.1080/07391102.2021.1874529) Epitope-Based Vaccine

Mohamed A. Soltan^1^*, Waleed K. Abdulsahib^2^, Mahmoud Amer^3^, Ahmed M Refaat^4^, Alaa A. Bagalagel^5^, Reem M. Diri^5^, Sarah Albogami^6^, Eman Fayad^6^, Refaat A. Eid^7^, Sherin M. A. Sharaf^8^, Sameh S. Elhady^9^, Khaled M. Darwish^10^ and Muhammad Alaa Eldeen^11^*

**Supplementary tables**

**Table S1.** Predicted scores of several physicochemical characteristics of the proposed vaccine.

| **Physicochemical Characteristic** | Molecular Weight | Theoretical pI | Extinction Coefficient | GRAVY | Aliphatic Index | Instability index |
| --- | --- | --- | --- | --- | --- | --- |
| **Score** | 37.04 kDa | 9.96 | 45420 M^-1^ cm^-1^ | -0.339 | 73.3 | 38.98 |

**Table S2.** Atom-atom interactions and bonding distances with representative diagrams for the depicted epitope/TLR-4 interfaces

| **Epitope (chain C) /TLR-4 protomer B (chain B)** | **Representative Diagram** ^a,b,c^ |
| --- | --- |
| **Hydrogen bonds**  **Atom Atom Res Res Atom Atom Res Res Distance**  **no. name name no. Chain no. name name no. Chain (Å)**  1. 14908 NH2 ARG 355 B <--> 21653 O GLY 167 C 2.71  2. 15317 NH2 ARG 382 B <--> 21318 O PHE 145 C 2.90  3. 15965 OE2 GLU 425 B <--> 22167 NZ LYS 201 C 2.74  4. 16015 OD2 ASP 428 B <--> 21359 N GLY 147 C 3.15  5. 16048 OE1 GLN 430 B <--> 21315 N PHE 145 C 2.65  6. 16048 OE1 GLN 430 B <--> 21335 N ARG 146 C 2.99  7. 16468 OG SER 455 B <--> 21305 OH TYR 144 C 2.76  8. 16805 NZ LYS 477 B <--> 21278 O ILE 143 C 2.74  9. 17159 OG1 THR 499 B <--> 21215 OG1 THR 139 C 3.04  10. 17213 OD2 ASP 502 B <--> 21010 NH1 ARG 125 C 2.70  11. 17213 OD2 ASP 502 B <--> 21011 NH2 ARG 125 C 3.20  12. 17525 OE1 GLN 523 B <--> 21201 OG1 THR 138 C 3.23  13. 18417 NE2 GLN 578 B <--> 20689 O SER 100 C 2.86  14. 18832 OE2 GLU 603 B <--> 21129 NE ARG 132 C 2.98  15. 18832 OE2 GLU 603 B <--> 21132 NH2 ARG 132 C 2.86  **Salt bridges**  **Atom Atom Res Res Atom Atom Res Res Distance**  **no. name name no. Chain no. name name no. Chain (Å)**  1. 15965 OE2 GLU 425 B <--> 22167 NZ LYS 201 C 2.74  2. 17213 OD2 ASP 502 B <--> 21010 NH1 ARG 125 C 2.70  3. 18831 OE1 GLU 603 B <--> 21132 NH2 ARG 132 C 2.86  **Non-bonded contacts**  **Atom Atom Res Res Atom Atom Res Res Distance**  **no. name name no. Chain no. name name no. Chain (Å)**  1. 9623 CH2 TRP 26 B <--> 20444 CE2 PHE 81 C 3.71  2. 9679 CG1 VAL 30 B <--> 20414 O MET 79 C 3.89  3. 9679 CG1 VAL 30 B <--> 20415 CB MET 79 C 3.73  4. 9679 CG1 VAL 30 B <--> 20417 SD MET 79 C 3.66  5. 9693 O GLU 31 B <--> 20414 O MET 79 C 3.10  6. 9693 O GLU 31 B <--> 20442 CD2 PHE 81 C 3.61  7. 9706 CA VAL 32 B <--> 20414 O MET 79 C 3.73  8. 9708 O VAL 32 B <--> 20429 CA GLY 80 C 3.46  9. 9711 CG2 VAL 32 B <--> 20414 O MET 79 C 3.88  10. 14906 CZ ARG 355 B <--> 21653 O GLY 167 C 3.50  11. 14907 NH1 ARG 355 B <--> 21653 O GLY 167 C 3.58  12. 14908 NH2 ARG 355 B <--> 21652 C GLY 167 C 3.78  13. 14908 NH2 ARG 355 B <--> 21653 O GLY 167 C 2.71  14. 14908 NH2 ARG 355 B <--> 21658 CA PRO 168 C 3.61  15. 14908 NH2 ARG 355 B <--> 21659 C PRO 168 C 3.02  16. 14908 NH2 ARG 355 B <--> 21660 O PRO 168 C 3.34  17. 14908 NH2 ARG 355 B <--> 21671 N GLY 169 C 3.06  18. 14908 NH2 ARG 355 B <--> 21672 CA GLY 169 C 3.46  19. 15235 CE2 PHE 377 B <--> 21639 O PRO 166 C 3.63  20. 15236 CZ PHE 377 B <--> 21653 O GLY 167 C 3.37  21. 15317 NH2 ARG 382 B <--> 21317 C PHE 145 C 3.66  22. 15317 NH2 ARG 382 B <--> 21318 O PHE 145 C 2.90  23. 15317 NH2 ARG 382 B <--> 21335 N ARG 146 C 3.82  24. 15317 NH2 ARG 382 B <--> 21336 CA ARG 146 C 3.53  25. 15624 CE1 TYR 403 B <--> 21683 CG PRO 170 C 3.75  26. 15624 CE1 TYR 403 B <--> 21684 CD PRO 170 C 3.77  27. 15627 OH TYR 403 B <--> 21371 CG PRO 148 C 3.82  28. 15627 OH TYR 403 B <--> 21640 CB PRO 166 C 3.51  29. 15706 CE1 PHE 408 B <--> 21317 C PHE 145 C 3.83  30. 15707 CE2 PHE 408 B <--> 20977 CA GLY 122 C 3.87  31. 15707 CE2 PHE 408 B <--> 20978 C GLY 122 C 3.60  32. 15707 CE2 PHE 408 B <--> 20979 O GLY 122 C 3.07  33. 15708 CZ PHE 408 B <--> 20979 O GLY 122 C 3.30  34. 15708 CZ PHE 408 B <--> 21316 CA PHE 145 C 3.75  35. 15963 CD GLU 425 B <--> 22167 NZ LYS 201 C 3.58  36. 15964 OE1 GLU 425 B <--> 22166 CE LYS 201 C 3.89  37. 15964 OE1 GLU 425 B <--> 22167 NZ LYS 201 C 3.57  38. 15965 OE2 GLU 425 B <--> 22166 CE LYS 201 C 3.46  39. 15965 OE2 GLU 425 B <--> 22167 NZ LYS 201 C 2.74  40. 15979 ND1 HSD 426 B <--> 21371 CG PRO 148 C 3.87  41. 15980 CE1 HSD 426 B <--> 21361 C GLY 147 C 3.64  42. 15980 CE1 HSD 426 B <--> 21362 O GLY 147 C 3.89  43. 15980 CE1 HSD 426 B <--> 21366 N PRO 148 C 3.44  44. 15980 CE1 HSD 426 B <--> 21367 CA PRO 148 C 3.75  45. 15980 CE1 HSD 426 B <--> 21371 CG PRO 148 C 3.59  46. 15980 CE1 HSD 426 B <--> 21372 CD PRO 148 C 3.52  47. 15981 NE2 HSD 426 B <--> 21366 N PRO 148 C 3.79  48. 15981 NE2 HSD 426 B <--> 21367 CA PRO 148 C 3.62  49. 16015 OD2 ASP 428 B <--> 21359 N GLY 147 C 3.15  50. 16015 OD2 ASP 428 B <--> 21360 CA GLY 147 C 3.32  51. 16046 CD GLN 430 B <--> 21300 CD1 TYR 144 C 3.87  52. 16046 CD GLN 430 B <--> 21315 N PHE 145 C 3.89  53. 16046 CD GLN 430 B <--> 21335 N ARG 146 C 3.87  54. 16048 OE1 GLN 430 B <--> 21295 CA TYR 144 C 3.22  55. 16048 OE1 GLN 430 B <--> 21296 C TYR 144 C 3.44  56. 16048 OE1 GLN 430 B <--> 21298 CB TYR 144 C 3.72  57. 16048 OE1 GLN 430 B <--> 21300 CD1 TYR 144 C 3.84  58. 16048 OE1 GLN 430 B <--> 21315 N PHE 145 C 2.65  59. 16048 OE1 GLN 430 B <--> 21316 CA PHE 145 C 3.54  60. 16048 OE1 GLN 430 B <--> 21317 C PHE 145 C 3.54  61. 16048 OE1 GLN 430 B <--> 21335 N ARG 146 C 2.99  62. 16064 ND1 HSD 431 B <--> 20936 CB HSD 119 C 3.42  63. 16064 ND1 HSD 431 B <--> 20937 CG HSD 119 C 3.63  64. 16064 ND1 HSD 431 B <--> 20939 ND1 HSD 119 C 3.87  65. 16065 CE1 HSD 431 B <--> 20936 CB HSD 119 C 3.61  66. 16401 CE2 TYR 451 B <--> 21362 O GLY 147 C 3.80  67. 16402 CZ TYR 451 B <--> 21362 O GLY 147 C 3.65  68. 16403 OH TYR 451 B <--> 21360 CA GLY 147 C 3.25  69. 16403 OH TYR 451 B <--> 21361 C GLY 147 C 3.79  70. 16403 OH TYR 451 B <--> 21362 O GLY 147 C 3.49  71. 16438 OD1 ASP 453 B <--> 21302 CE1 TYR 144 C 3.81  72. 16439 OD2 ASP 453 B <--> 21300 CD1 TYR 144 C 3.84  73. 16439 OD2 ASP 453 B <--> 21302 CE1 TYR 144 C 3.38  74. 16467 CB SER 455 B <--> 21302 CE1 TYR 144 C 3.24  75. 16467 CB SER 455 B <--> 21304 CZ TYR 144 C 3.50  76. 16467 CB SER 455 B <--> 21305 OH TYR 144 C 3.39  77. 16468 OG SER 455 B <--> 21302 CE1 TYR 144 C 3.30  78. 16468 OG SER 455 B <--> 21304 CZ TYR 144 C 3.33  79. 16468 OG SER 455 B <--> 21305 OH TYR 144 C 2.76  80. 16513 CE1 HSD 458 B <--> 20902 O TYR 117 C 3.60  81. 16514 NE2 HSD 458 B <--> 20902 O TYR 117 C 3.90  82. 16514 NE2 HSD 458 B <--> 20903 CB TYR 117 C 3.56  83. 16768 CG2 VAL 475 B <--> 21215 OG1 THR 139 C 3.84  84. 16804 CE LYS 477 B <--> 21264 O PRO 142 C 3.49  85. 16804 CE LYS 477 B <--> 21302 CE1 TYR 144 C 3.69  86. 16804 CE LYS 477 B <--> 21304 CZ TYR 144 C 3.76  87. 16804 CE LYS 477 B <--> 21305 OH TYR 144 C 3.70  88. 16805 NZ LYS 477 B <--> 21263 C PRO 142 C 3.81  89. 16805 NZ LYS 477 B <--> 21264 O PRO 142 C 3.45  90. 16805 NZ LYS 477 B <--> 21277 C ILE 143 C 3.79  91. 16805 NZ LYS 477 B <--> 21278 O ILE 143 C 2.74  92. 16805 NZ LYS 477 B <--> 21300 CD1 TYR 144 C 3.83  93. 16805 NZ LYS 477 B <--> 21302 CE1 TYR 144 C 3.37  94. 16840 CB ALA 479 B <--> 21305 OH TYR 144 C 3.30  95. 17157 CB THR 499 B <--> 21215 OG1 THR 139 C 3.62  96. 17159 OG1 THR 499 B <--> 21215 OG1 THR 139 C 3.04  97. 17171 CB PHE 500 B <--> 21214 CG2 THR 139 C 3.80  98. 17211 CG ASP 502 B <--> 21305 OH TYR 144 C 3.90  99. 17213 OD2 ASP 502 B <--> 21009 CZ ARG 125 C 3.39  100. 17213 OD2 ASP 502 B <--> 21010 NH1 ARG 125 C 2.70  101. 17213 OD2 ASP 502 B <--> 21011 NH2 ARG 125 C 3.20  102. 17213 OD2 ASP 502 B <--> 21305 OH TYR 144 C 3.42  103. 17242 OG SER 504 B <--> 21011 NH2 ARG 125 C 3.37  104. 17252 CB GLN 505 B <--> 20910 OH TYR 117 C 3.88  105. 17256 OE1 GLN 505 B <--> 20882 CB ALA 115 C 3.74  106. 17256 OE1 GLN 505 B <--> 20907 CE1 TYR 117 C 3.64  107. 17283 NE2 GLN 507 B <--> 20910 OH TYR 117 C 3.47  108. 17525 OE1 GLN 523 B <--> 21197 C THR 138 C 3.89  109. 17525 OE1 GLN 523 B <--> 21198 O THR 138 C 3.71  110. 17525 OE1 GLN 523 B <--> 21199 CB THR 138 C 3.69  111. 17525 OE1 GLN 523 B <--> 21201 OG1 THR 138 C 3.23  112. 17525 OE1 GLN 523 B <--> 21213 CB THR 139 C 3.84  113. 17575 ND2 ASN 526 B <--> 21011 NH2 ARG 125 C 3.31  114. 17925 CG1 VAL 548 B <--> 21149 O HSD 133 C 3.55  115. 17925 CG1 VAL 548 B <--> 21165 C GLY 134 C 3.77  116. 17925 CG1 VAL 548 B <--> 21170 N GLY 135 C 3.81  117. 17926 CG2 VAL 548 B <--> 21164 CA GLY 134 C 3.88  118. 17926 CG2 VAL 548 B <--> 21165 C GLY 134 C 3.79  119. 17926 CG2 VAL 548 B <--> 21166 O GLY 134 C 3.44  120. 18006 CD2 LEU 553 B <--> 20680 OG SER 99 C 3.31  121. 18328 CB PHE 573 B <--> 21149 O HSD 133 C 3.65  122. 18330 CD1 PHE 573 B <--> 21125 O ARG 132 C 3.37  123. 18330 CD1 PHE 573 B <--> 21148 C HSD 133 C 3.79  124. 18330 CD1 PHE 573 B <--> 21163 N GLY 134 C 3.78  125. 18332 CE1 PHE 573 B <--> 21111 O ASN 131 C 3.69  126. 18332 CE1 PHE 573 B <--> 21124 C ARG 132 C 3.85  127. 18332 CE1 PHE 573 B <--> 21125 O ARG 132 C 3.27  128. 18416 CD GLN 578 B <--> 20689 O SER 100 C 3.81  129. 18418 OE1 GLN 578 B <--> 20716 N SER 102 C 3.42  130. 18418 OE1 GLN 578 B <--> 20720 CB SER 102 C 3.75  131. 18417 NE2 GLN 578 B <--> 20689 O SER 100 C 2.86  132. 18814 CG2 VAL 602 B <--> 21125 O ARG 132 C 3.60  133. 18829 CG GLU 603 B <--> 21129 NE ARG 132 C 3.72  134. 18829 CG GLU 603 B <--> 21132 NH2 ARG 132 C 3.73  135. 18830 CD GLU 603 B <--> 21129 NE ARG 132 C 3.39  136. 18830 CD GLU 603 B <--> 21132 NH2 ARG 132 C 3.64  137. 18832 OE2 GLU 603 B <--> 21129 NE ARG 132 C 2.98  138. 18832 OE2 GLU 603 B <--> 21130 CZ ARG 132 C 3.31  139. 18832 OE2 GLU 603 B <--> 21132 NH2 ARG 132 C 2.86  140. 18877 NE ARG 606 B <--> 20721 OG SER 102 C 3.65  141. 18878 CZ ARG 606 B <--> 20721 OG SER 102 C 3.62  142. 18879 NH1 ARG 606 B <--> 20720 CB SER 102 C 3.52  143. 18879 NH1 ARG 606 B <--> 20721 OG SER 102 C 3.54  144. 18880 NH2 ARG 606 B <--> 20719 O SER 102 C 3.68 | 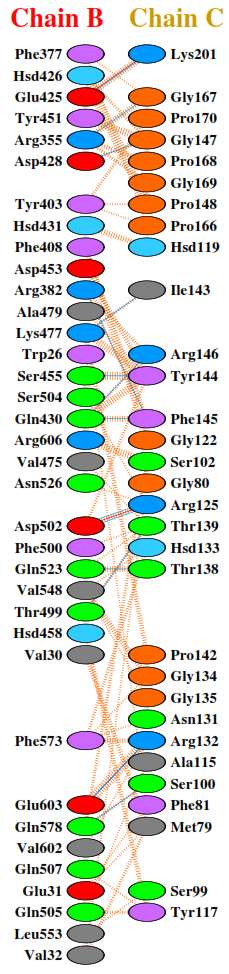 |
| **Epitope (chain C) /TLR-4 protomer A (chain A)** | **Representative Diagram** ^a,b,c^ |
| **Hydrogen bonds**  **Atom Atom Res Res Atom Atom Res Res Distance**  **no. name name no. Chain no. name name no. Chain (Å)**  1. 6495 NZ LYS 435 A <--> 20941 NE2 HSD 119 C 3.08  2. 6509 N GLN 436 A <--> 20926 OD1 ASP 118 C 3.01  3. 6543 N SER 438 A <--> 20927 OD2 ASP 118 C 2.70  4. 6548 OG SER 438 A <--> 20927 OD2 ASP 118 C 3.21  5. 6975 N ASN 464 A <--> 20224 O LYS 65 C 2.87  6. 7291 OE1 GLU 485 A <--> 20657 N GLY 97 C 2.81  **Non-bonded contacts**  **Atom Atom Res Res Atom Atom Res Res Distance**  **no. name name no. Chain no. name name no. Chain (Å)**  1. 6488 CA LYS 435 A <--> 20926 OD1 ASP 118 C 3.77  2. 6489 C LYS 435 A <--> 20926 OD1 ASP 118 C 3.85  3. 6493 CD LYS 435 A <--> 20941 NE2 HSD 119 C 3.77  4. 6494 CE LYS 435 A <--> 20938 CD2 HSD 119 C 3.77  5. 6494 CE LYS 435 A <--> 20941 NE2 HSD 119 C 3.46  6. 6495 NZ LYS 435 A <--> 20938 CD2 HSD 119 C 3.83  7. 6495 NZ LYS 435 A <--> 20941 NE2 HSD 119 C 3.08  8. 6509 N GLN 436 A <--> 20926 OD1 ASP 118 C 3.01  9. 6510 CA GLN 436 A <--> 20926 OD1 ASP 118 C 3.79  10. 6526 N MET 437 A <--> 20925 CG ASP 118 C 3.75  11. 6526 N MET 437 A <--> 20926 OD1 ASP 118 C 3.47  12. 6526 N MET 437 A <--> 20927 OD2 ASP 118 C 3.29  13. 6527 CA MET 437 A <--> 20927 OD2 ASP 118 C 3.82  14. 6528 C MET 437 A <--> 20927 OD2 ASP 118 C 3.67  15. 6543 N SER 438 A <--> 20925 CG ASP 118 C 3.77  16. 6543 N SER 438 A <--> 20927 OD2 ASP 118 C 2.70  17. 6544 CA SER 438 A <--> 20927 OD2 ASP 118 C 3.37  18. 6547 CB SER 438 A <--> 20927 OD2 ASP 118 C 3.17  19. 6547 CB SER 438 A <--> 20959 CZ PHE 120 C 3.87  20. 6548 OG SER 438 A <--> 20924 CB ASP 118 C 3.76  21. 6548 OG SER 438 A <--> 20927 OD2 ASP 118 C 3.21  22. 6559 CG GLU 439 A <--> 20955 CD1 PHE 120 C 3.89  23. 6562 OE2 GLU 439 A <--> 20950 CA PHE 120 C 3.66  24. 6562 OE2 GLU 439 A <--> 20951 C PHE 120 C 3.80  25. 6562 OE2 GLU 439 A <--> 20969 N GLY 121 C 3.53  26. 6570 CA PHE 440 A <--> 20267 CB ALA 68 C 3.79  27. 6908 O ARG 460 A <--> 20924 CB ASP 118 C 3.43  28. 6909 CB ARG 460 A <--> 20920 N ASP 118 C 3.67  29. 6910 CG ARG 460 A <--> 20920 N ASP 118 C 3.60  30. 6914 NH1 ARG 460 A <--> 20881 O ALA 115 C 3.70  31. 6961 CD1 PHE 463 A <--> 20224 O LYS 65 C 3.12  32. 6963 CE1 PHE 463 A <--> 20224 O LYS 65 C 3.48  33. 6963 CE1 PHE 463 A <--> 20255 C ALA 67 C 3.60  34. 6963 CE1 PHE 463 A <--> 20256 O ALA 67 C 3.41  35. 6963 CE1 PHE 463 A <--> 20263 N ALA 68 C 3.66  36. 6963 CE1 PHE 463 A <--> 20264 CA ALA 68 C 3.69  37. 6965 CZ PHE 463 A <--> 20264 CA ALA 68 C 3.88  38. 6975 N ASN 464 A <--> 20223 C LYS 65 C 3.80  39. 6975 N ASN 464 A <--> 20224 O LYS 65 C 2.87  40. 6976 CA ASN 464 A <--> 20224 O LYS 65 C 3.63  41. 6977 C ASN 464 A <--> 20244 CA ALA 66 C 3.78  42. 6979 CB ASN 464 A <--> 20223 C LYS 65 C 3.79  43. 6979 CB ASN 464 A <--> 20224 O LYS 65 C 3.53  44. 6979 CB ASN 464 A <--> 20243 N ALA 66 C 3.84  45. 6979 CB ASN 464 A <--> 20244 CA ALA 66 C 3.59  46. 6979 CB ASN 464 A <--> 20247 CB ALA 66 C 3.69  47. 6982 OD1 ASN 464 A <--> 20173 CE3 TRP 62 C 3.75  48. 6982 OD1 ASN 464 A <--> 20225 CB LYS 65 C 3.79  49. 6981 ND2 ASN 464 A <--> 20167 O TRP 62 C 3.59  50. 6989 N GLY 465 A <--> 20224 O LYS 65 C 3.78  51. 6989 N GLY 465 A <--> 20244 CA ALA 66 C 3.70  52. 6989 N GLY 465 A <--> 20246 O ALA 66 C 3.85  53. 6990 CA GLY 465 A <--> 20246 O ALA 66 C 3.30  54. 7041 ND2 ASN 468 A <--> 20246 O ALA 66 C 3.14  55. 7274 NE2 GLN 484 A <--> 20657 N GLY 97 C 3.90  56. 7288 CB GLU 485 A <--> 20651 CA GLY 96 C 3.78  57. 7290 CD GLU 485 A <--> 20657 N GLY 97 C 3.86  58. 7291 OE1 GLU 485 A <--> 20652 C GLY 96 C 3.80  59. 7291 OE1 GLU 485 A <--> 20657 N GLY 97 C 2.81  60. 7291 OE1 GLU 485 A <--> 20658 CA GLY 97 C 3.55  61. 7317 CB PHE 487 A <--> 20645 C GLY 95 C 3.66  62. 7317 CB PHE 487 A <--> 20650 N GLY 96 C 3.56  63. 7318 CG PHE 487 A <--> 20644 CA GLY 95 C 3.68  64. 7318 CG PHE 487 A <--> 20645 C GLY 95 C 3.65  65. 7318 CG PHE 487 A <--> 20650 N GLY 96 C 3.56  66. 7319 CD1 PHE 487 A <--> 20644 CA GLY 95 C 3.63  67. 7320 CD2 PHE 487 A <--> 20650 N GLY 96 C 3.40  68. 7321 CE1 PHE 487 A <--> 20644 CA GLY 95 C 3.55  69. 7322 CE2 PHE 487 A <--> 20174 NE1 TRP 62 C 3.76  70. 7322 CE2 PHE 487 A <--> 20172 CE2 TRP 62 C 3.58  71. 7322 CE2 PHE 487 A <--> 20175 CZ2 TRP 62 C 3.75  72. 7322 CE2 PHE 487 A <--> 20626 O PHE 94 C 3.75  73. 7323 CZ PHE 487 A <--> 20644 CA GLY 95 C 3.81 | 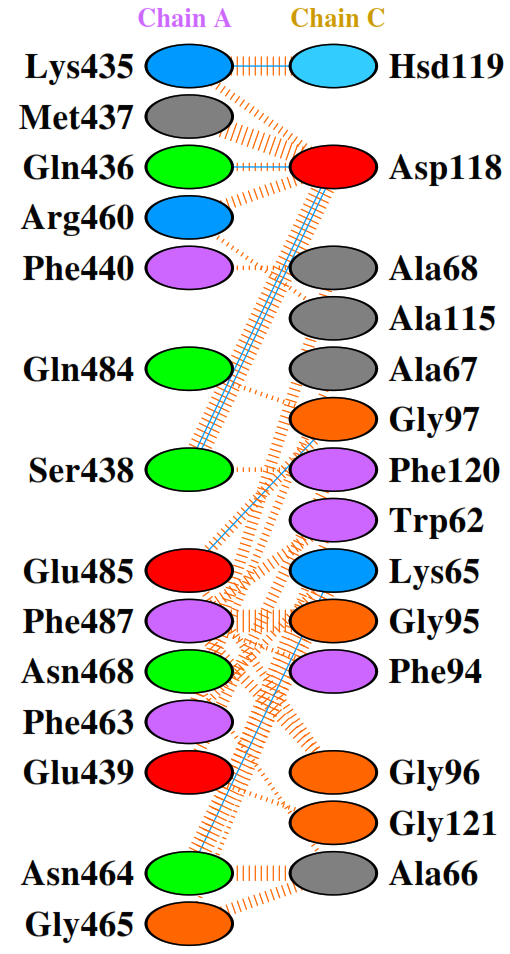 |

^a^ *The number of H-bond lines between any two residues indicates the number of potential hydrogen bonds between them. For non-bonded contacts, which can be plentiful, width of the striped line is proportional to the number of atomic contacts.*

^b^ *Residue colours: Positive (H,K,R); negative (D,E); S,T,N,Q = neutral; A,V,L,I,M = aliphatic; F,Y,W = aromatic; P,G = Pro&Gly; C = cysteine.*

^c^ *Interaction bonding key:*


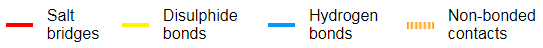


**Table S3.** Atom-atom interactions and bonding distances with representative diagrams for the depicted epitope/TLR-8 interfaces

| **Epitope (chain C) /TLR-8 protomer B (chain B)** | **Representative Diagram** ^a,b,c^ |
| --- | --- |
| **Hydrogen bonds**  **Atom Atom Res Res Atom Atom Res Res Distance**  **no. name name no. Chain no. name name no. Chain (Å)**  1. 12594 OG SER 71 B <--> 23914 OG1 THR 10 C 2.82  2. 12991 ND1 HSD 96 B <--> 23911 O THR 10 C 3.00  3. 13420 OD2 ASP 134 B <--> 23966 NZ LYS 13 C 2.80  4. 14898 NZ LYS 226 B <--> 23788 OE2 GLU 1 C 2.76  5. 18365 NE2 HSD 464 B <--> 24040 NE ARG 17 C 3.06  6. 18365 NE2 HSD 464 B <--> 24043 NH2 ARG 17 C 3.30  7. 18574 NZ LYS 476 B <--> 24276 OE2 GLU 32 C 2.75  8. 19476 OD2 ASP 536 B <--> 24409 NH2 ARG 41 C 2.90  9. 19840 OD2 ASP 560 B <--> 24406 NE ARG 41 C 2.95  10. 19840 OD2 ASP 560 B <--> 24409 NH2 ARG 41 C 2.77  11. 19840 OD2 ASP 560 B <--> 24462 NZ LYS 44 C 2.66  12. 20696 OE1 GLU 612 B <--> 24532 NH2 ARG 48 C 3.24  13. 21252 OD1 ASP 645 B <--> 24440 NH2 ARG 43 C 2.83  14. 21253 OD2 ASP 645 B <--> 24437 NE ARG 43 C 2.93  15. 21282 OG SER 647 B <--> 24440 NH2 ARG 43 C 2.89  16. 21622 OE1 GLU 668 B <--> 24507 NH1 ARG 47 C 2.89  17. 22032 OE2 GLU 691 B <--> 25373 OH TYR 107 C 2.73  **Salt bridges**  **Atom Atom Res Res Atom Atom Res Res Distance**  **no. name name no. Chain no. name name no. Chain (Å)**  1. 13420 OD2 ASP 134 B <--> 23966 NZ LYS 13 C 2.80  2. 14898 NZ LYS 226 B <--> 23787 OE1 GLU 1 C 2.76  3. 18574 NZ LYS 476 B <--> 24276 OE2 GLU 32 C 2.75  4. 19476 OD2 ASP 536 B <--> 24409 NH2 ARG 41 C 2.90  5. 19840 OD2 ASP 560 B <--> 24409 NH2 ARG 41 C 2.77  6. 19840 OD2 ASP 560 B <--> 24462 NZ LYS 44 C 2.66  7. 20697 OE2 GLU 612 B <--> 24531 NH1 ARG 48 C 3.05  8. 21252 OD1 ASP 645 B <--> 24440 NH2 ARG 43 C 2.83  9. 21622 OE1 GLU 668 B <--> 24507 NH1 ARG 47 C 2.89  **Non-bonded contacts**  **Atom Atom Res Res Atom Atom Res Res Distance**  **no. name name no. Chain no. name name no. Chain (Å)**  1. 12593 CB SER 71 B <--> 23914 OG1 THR 10 C 3.32  2. 12594 OG SER 71 B <--> 23912 CB THR 10 C 3.63  3. 12594 OG SER 71 B <--> 23914 OG1 THR 10 C 2.82  4. 12594 OG SER 71 B <--> 23913 CG2 THR 10 C 3.68  5. 12976 ND2 ASN 95 B <--> 23862 CG2 ILE 7 C 3.60  6. 12991 ND1 HSD 96 B <--> 23911 O THR 10 C 3.00  7. 12992 CE1 HSD 96 B <--> 23910 C THR 10 C 3.73  8. 12992 CE1 HSD 96 B <--> 23911 O THR 10 C 3.12  9. 12992 CE1 HSD 96 B <--> 23923 CA LEU 11 C 3.88  10. 13333 OE1 GLU 129 B <--> 23811 CB ALA 3 C 3.78  11. 13366 CD1 LEU 131 B <--> 23862 CG2 ILE 7 C 3.46  12. 13420 OD2 ASP 134 B <--> 23965 CE LYS 13 C 3.85  13. 13420 OD2 ASP 134 B <--> 23966 NZ LYS 13 C 2.80  14. 13657 OE2 GLU 150 B <--> 23811 CB ALA 3 C 3.67  15. 14896 CD LYS 226 B <--> 23788 OE2 GLU 1 C 3.41  16. 14897 CE LYS 226 B <--> 23788 OE2 GLU 1 C 3.55  17. 14898 NZ LYS 226 B <--> 23786 CD GLU 1 C 3.11  18. 14898 NZ LYS 226 B <--> 23787 OE1 GLU 1 C 2.90  19. 14898 NZ LYS 226 B <--> 23788 OE2 GLU 1 C 2.76  20. 18364 CE1 HSD 464 B <--> 24040 NE ARG 17 C 3.89  21. 18364 CE1 HSD 464 B <--> 24043 NH2 ARG 17 C 3.68  22. 18365 NE2 HSD 464 B <--> 24040 NE ARG 17 C 3.06  23. 18365 NE2 HSD 464 B <--> 24041 CZ ARG 17 C 3.66  24. 18365 NE2 HSD 464 B <--> 24043 NH2 ARG 17 C 3.30  25. 18519 CG PRO 473 B <--> 24206 CB CYS 28 C 3.90  26. 18570 CB LYS 476 B <--> 24250 CB LYS 31 C 3.71  27. 18572 CD LYS 476 B <--> 24273 CG GLU 32 C 3.77  28. 18572 CD LYS 476 B <--> 24276 OE2 GLU 32 C 3.28  29. 18573 CE LYS 476 B <--> 24276 OE2 GLU 32 C 2.99  30. 18574 NZ LYS 476 B <--> 24274 CD GLU 32 C 3.90  31. 18574 NZ LYS 476 B <--> 24276 OE2 GLU 32 C 2.75  32. 18592 CB PRO 477 B <--> 24207 SG CYS 28 C 3.85  33. 18593 CG PRO 477 B <--> 24204 C CYS 28 C 3.75  34. 18593 CG PRO 477 B <--> 24205 O CYS 28 C 3.71  35. 18593 CG PRO 477 B <--> 24207 SG CYS 28 C 3.67  36. 18593 CG PRO 477 B <--> 24213 N LEU 29 C 3.82  37. 18593 CG PRO 477 B <--> 24214 CA LEU 29 C 3.77  38. 18593 CG PRO 477 B <--> 24220 CD2 LEU 29 C 3.72  39. 18594 CD PRO 477 B <--> 24205 O CYS 28 C 3.67  40. 18609 NE2 GLN 478 B <--> 24216 O LEU 29 C 3.57  41. 18609 NE2 GLN 478 B <--> 24220 CD2 LEU 29 C 3.79  42. 18609 NE2 GLN 478 B <--> 24273 CG GLU 32 C 3.89  43. 19440 OH TYR 534 B <--> 24276 OE2 GLU 32 C 3.86  44. 19474 CG ASP 536 B <--> 24409 NH2 ARG 41 C 3.42  45. 19475 OD1 ASP 536 B <--> 24409 NH2 ARG 41 C 3.37  46. 19476 OD2 ASP 536 B <--> 24409 NH2 ARG 41 C 2.90  47. 19506 OG1 THR 538 B <--> 24407 CZ ARG 41 C 3.53  48. 19506 OG1 THR 538 B <--> 24408 NH1 ARG 41 C 3.26  49. 19506 OG1 THR 538 B <--> 24409 NH2 ARG 41 C 3.35  50. 19505 CG2 THR 538 B <--> 24407 CZ ARG 41 C 3.67  51. 19505 CG2 THR 538 B <--> 24409 NH2 ARG 41 C 3.47  52. 19838 CG ASP 560 B <--> 24406 NE ARG 41 C 3.66  53. 19838 CG ASP 560 B <--> 24409 NH2 ARG 41 C 3.43  54. 19838 CG ASP 560 B <--> 24462 NZ LYS 44 C 3.73  55. 19839 OD1 ASP 560 B <--> 24406 NE ARG 41 C 3.73  56. 19840 OD2 ASP 560 B <--> 24406 NE ARG 41 C 2.95  57. 19840 OD2 ASP 560 B <--> 24407 CZ ARG 41 C 3.30  58. 19840 OD2 ASP 560 B <--> 24409 NH2 ARG 41 C 2.77  59. 19840 OD2 ASP 560 B <--> 24461 CE LYS 44 C 3.22  60. 19840 OD2 ASP 560 B <--> 24462 NZ LYS 44 C 2.66  61. 19884 CE2 TYR 563 B <--> 24405 CD ARG 41 C 3.71  62. 19886 OH TYR 563 B <--> 24367 CB CYS 38 C 3.23  63. 19886 OH TYR 563 B <--> 24368 SG CYS 38 C 3.59  64. 19886 OH TYR 563 B <--> 24403 CB ARG 41 C 3.61  65. 19886 OH TYR 563 B <--> 24405 CD ARG 41 C 3.49  66. 20301 CG1 VAL 588 B <--> 24532 NH2 ARG 48 C 3.76  67. 20302 CG2 VAL 588 B <--> 24532 NH2 ARG 48 C 3.47  68. 20338 OD1 ASN 590 B <--> 24459 CG LYS 44 C 3.37  69. 20338 OD1 ASN 590 B <--> 24460 CD LYS 44 C 3.47  70. 20338 OD1 ASN 590 B <--> 24461 CE LYS 44 C 3.49  71. 20369 OG SER 592 B <--> 24389 CB THR 40 C 3.80  72. 20369 OG SER 592 B <--> 24390 CG2 THR 40 C 3.71  73. 20695 CD GLU 612 B <--> 24531 NH1 ARG 48 C 3.43  74. 20695 CD GLU 612 B <--> 24532 NH2 ARG 48 C 3.89  75. 20696 OE1 GLU 612 B <--> 24530 CZ ARG 48 C 3.66  76. 20696 OE1 GLU 612 B <--> 24531 NH1 ARG 48 C 3.16  77. 20696 OE1 GLU 612 B <--> 24532 NH2 ARG 48 C 3.24  78. 20697 OE2 GLU 612 B <--> 24458 CB LYS 44 C 3.70  79. 20697 OE2 GLU 612 B <--> 24531 NH1 ARG 48 C 3.05  80. 20728 CG1 VAL 614 B <--> 24390 CG2 THR 40 C 3.45  81. 20763 CB SER 616 B <--> 24391 OG1 THR 40 C 3.58  82. 20764 OG SER 616 B <--> 24389 CB THR 40 C 3.42  83. 20764 OG SER 616 B <--> 24391 OG1 THR 40 C 3.21  84. 20764 OG SER 616 B <--> 24390 CG2 THR 40 C 3.77  85. 21210 NE ARG 643 B <--> 24503 CG ARG 47 C 3.79  86. 21212 NH1 ARG 643 B <--> 24458 CB LYS 44 C 3.79  87. 21251 CG ASP 645 B <--> 24440 NH2 ARG 43 C 3.41  88. 21252 OD1 ASP 645 B <--> 24438 CZ ARG 43 C 3.79  89. 21252 OD1 ASP 645 B <--> 24440 NH2 ARG 43 C 2.83  90. 21253 OD2 ASP 645 B <--> 24390 CG2 THR 40 C 3.86  91. 21253 OD2 ASP 645 B <--> 24437 NE ARG 43 C 2.93  92. 21253 OD2 ASP 645 B <--> 24438 CZ ARG 43 C 3.36  93. 21253 OD2 ASP 645 B <--> 24440 NH2 ARG 43 C 3.14  94. 21281 CB SER 647 B <--> 24440 NH2 ARG 43 C 3.37  95. 21282 OG SER 647 B <--> 24391 OG1 THR 40 C 3.33  96. 21282 OG SER 647 B <--> 24440 NH2 ARG 43 C 2.89  97. 21294 CD1 LEU 648 B <--> 24391 OG1 THR 40 C 3.75  98. 21621 CD GLU 668 B <--> 24507 NH1 ARG 47 C 3.51  99. 21622 OE1 GLU 668 B <--> 24503 CG ARG 47 C 3.03  100. 21622 OE1 GLU 668 B <--> 24504 CD ARG 47 C 3.47  101. 21622 OE1 GLU 668 B <--> 24506 CZ ARG 47 C 3.84  102. 21622 OE1 GLU 668 B <--> 24507 NH1 ARG 47 C 2.89  103. 21656 ND1 HSD 670 B <--> 24436 CD ARG 43 C 3.79  104. 21656 ND1 HSD 670 B <--> 24437 NE ARG 43 C 3.76  105. 21657 CE1 HSD 670 B <--> 24436 CD ARG 43 C 3.87  106. 21690 CG ASN 672 B <--> 24440 NH2 ARG 43 C 3.88  107. 21692 OD1 ASN 672 B <--> 24440 NH2 ARG 43 C 3.82  108. 21691 ND2 ASN 672 B <--> 24438 CZ ARG 43 C 3.50  109. 21691 ND2 ASN 672 B <--> 24439 NH1 ARG 43 C 3.28  110. 21691 ND2 ASN 672 B <--> 24440 NH2 ARG 43 C 3.17  111. 21706 OD2 ASP 673 B <--> 24379 OG SER 39 C 3.88  112. 21970 O PRO 688 B <--> 25369 CD2 TYR 107 C 3.20  113. 21970 O PRO 688 B <--> 25371 CE2 TYR 107 C 3.84  114. 21982 CA ARG 689 B <--> 25369 CD2 TYR 107 C 3.56  115. 21982 CA ARG 689 B <--> 25371 CE2 TYR 107 C 3.37  116. 22029 CG GLU 691 B <--> 25373 OH TYR 107 C 3.77  117. 22030 CD GLU 691 B <--> 25373 OH TYR 107 C 3.50  118. 22032 OE2 GLU 691 B <--> 25372 CZ TYR 107 C 3.87  119. 22032 OE2 GLU 691 B <--> 25373 OH TYR 107 C 2.73  120. 22045 CD1 LEU 692 B <--> 24507 NH1 ARG 47 C 3.66  121. 22118 NH2 ARG 696 B <--> 24439 NH1 ARG 43 C 3.54  122. 22310 C SER 708 B <--> 25026 CZ PHE 81 C 3.79  123. 22311 O SER 708 B <--> 25025 CE2 PHE 81 C 3.44  124. 22311 O SER 708 B <--> 25026 CZ PHE 81 C 3.42  125. 22312 CB SER 708 B <--> 25026 CZ PHE 81 C 3.76  126. 22325 OD1 ASP 709 B <--> 25026 CZ PHE 81 C 3.90  127. 22369 CB SER 712 B <--> 25025 CE2 PHE 81 C 3.72  128. 22370 OG SER 712 B <--> 25018 C PHE 81 C 3.86  129. 22370 OG SER 712 B <--> 25019 O PHE 81 C 3.45  130. 22370 OG SER 712 B <--> 25023 CD2 PHE 81 C 3.56  131. 22370 OG SER 712 B <--> 25025 CE2 PHE 81 C 3.53  132. 22370 OG SER 712 B <--> 25036 N GLY 82 C 3.88  133. 22370 OG SER 712 B <--> 25037 CA GLY 82 C 3.77  134. 22381 OG SER 713 B <--> 25369 CD2 TYR 107 C 3.61  135. 22416 NH2 ARG 715 B <--> 25391 CA GLY 109 C 3.74 | 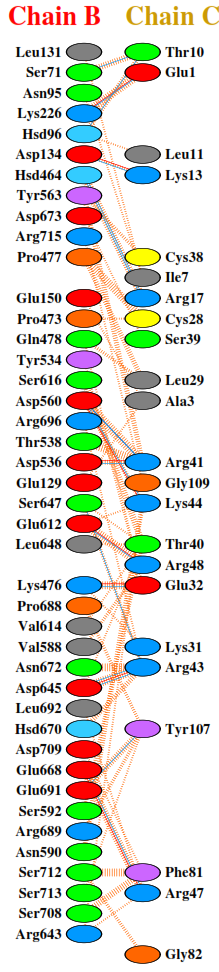 |
| **Epitope (chain C) /TLR-4 protomer A (chain A)** | **Representative Diagram** ^a,b,c^ |
| **Non-bonded contacts**  **Atom Atom Res Res Atom Atom Res Res Distance**  **no. name name no. Chain no. name name no. Chain (Å)**  1. 8019 O ARG 569 A <--> 24026 CB CYS 16 C 3.56  2. 8483 CD2 TYR 597 A <--> 24009 CE1 TYR 15 C 3.62  3. 8485 CE2 TYR 597 A <--> 24007 CD1 TYR 15 C 3.72  4. 8485 CE2 TYR 597 A <--> 24009 CE1 TYR 15 C 3.39  5. 9366 CZ ARG 650 A <--> 23989 CE2 TYR 14 C 3.80  6. 9367 NH1 ARG 650 A <--> 23989 CE2 TYR 14 C 3.42  7. 9368 NH2 ARG 650 A <--> 23991 OH TYR 14 C 3.75 | 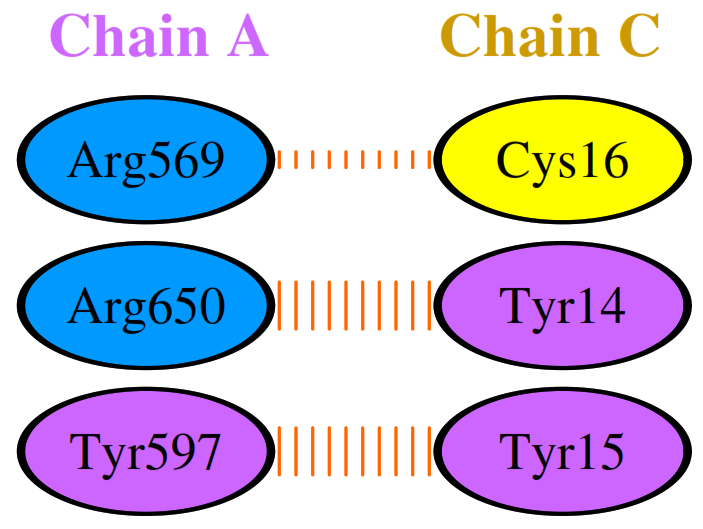 |

^a^ *The number of H-bond lines between any two residues indicates the number of potential hydrogen bonds between them. For non-bonded contacts, which can be plentiful, width of the striped line is proportional to the number of atomic contacts.*

^b^ *Residue colours: Positive (H,K,R); negative (D,E); S,T,N,Q = neutral; A,V,L,I,M = aliphatic; F,Y,W = aromatic; P,G = Pro&Gly; C = cysteine.*

^c^ *Interaction bonding key:*


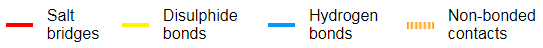


**Supplementary figures**


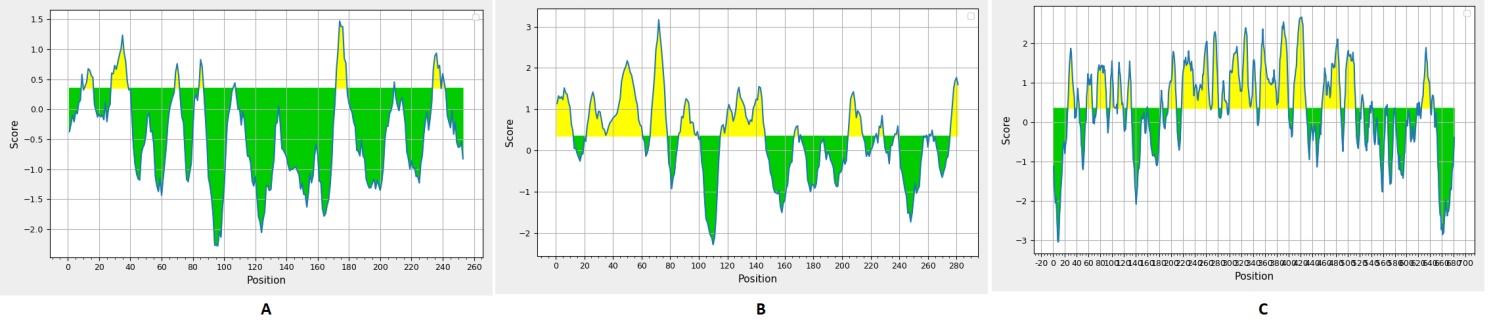


**Supplementary figure 1.** Bepipred linear epitope prediction for VP24 (A), VP30 (B), and envelope glycoprotein (C), the yellow section represents the B epitope part of each protein.


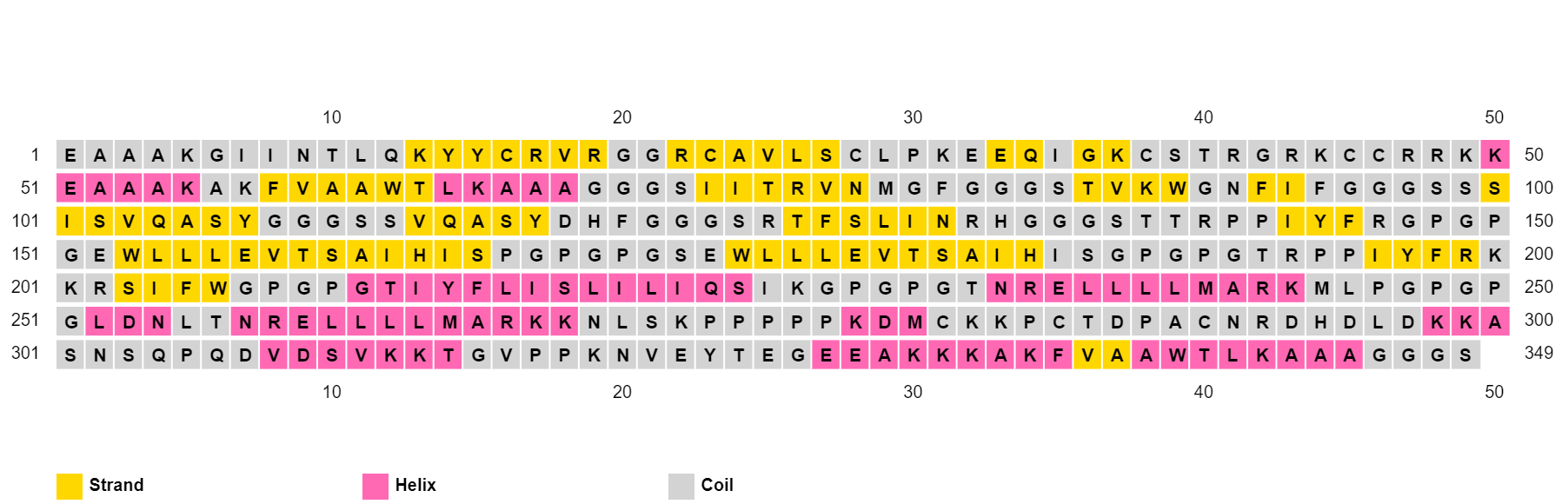


**Supplementary figure 2.** Secondary structure prediction of designed multitope vaccine using PESIPRED server.


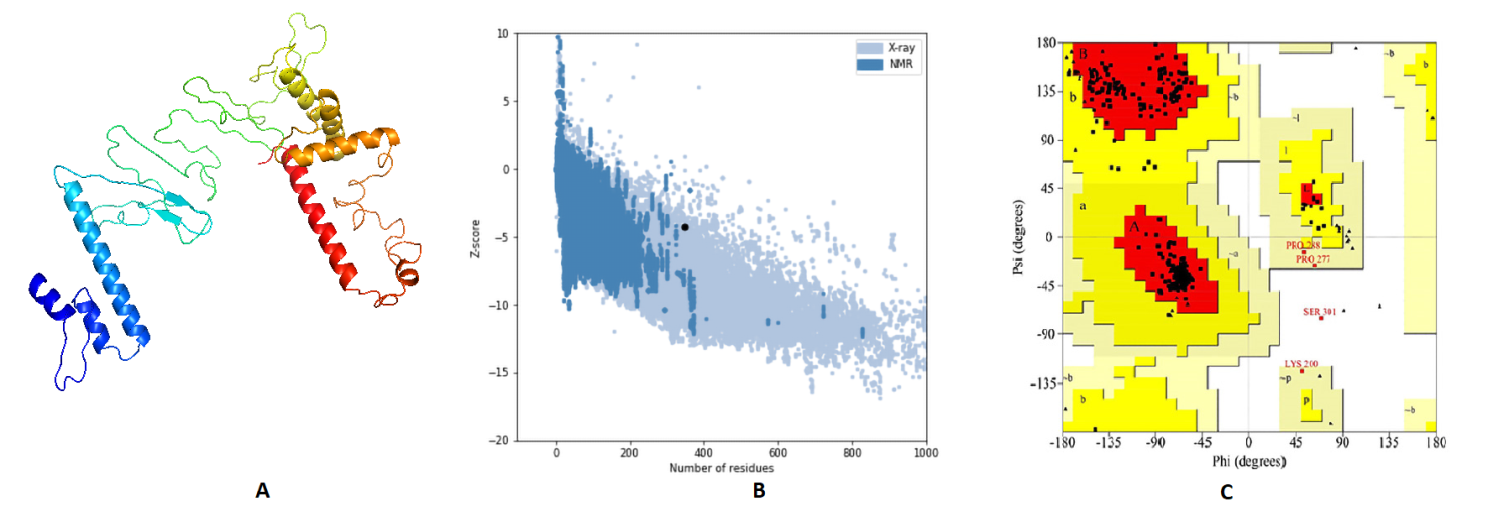


**Supplementary figure3.** Structural analysis of the predicted vaccine 3D structure. (**A**) The 3D structure of designed vaccine after refinement; (**B**) ProSA-web evaluation of the refined vaccine; (**C**) Ramachandran plot analysis of the refined vaccine.


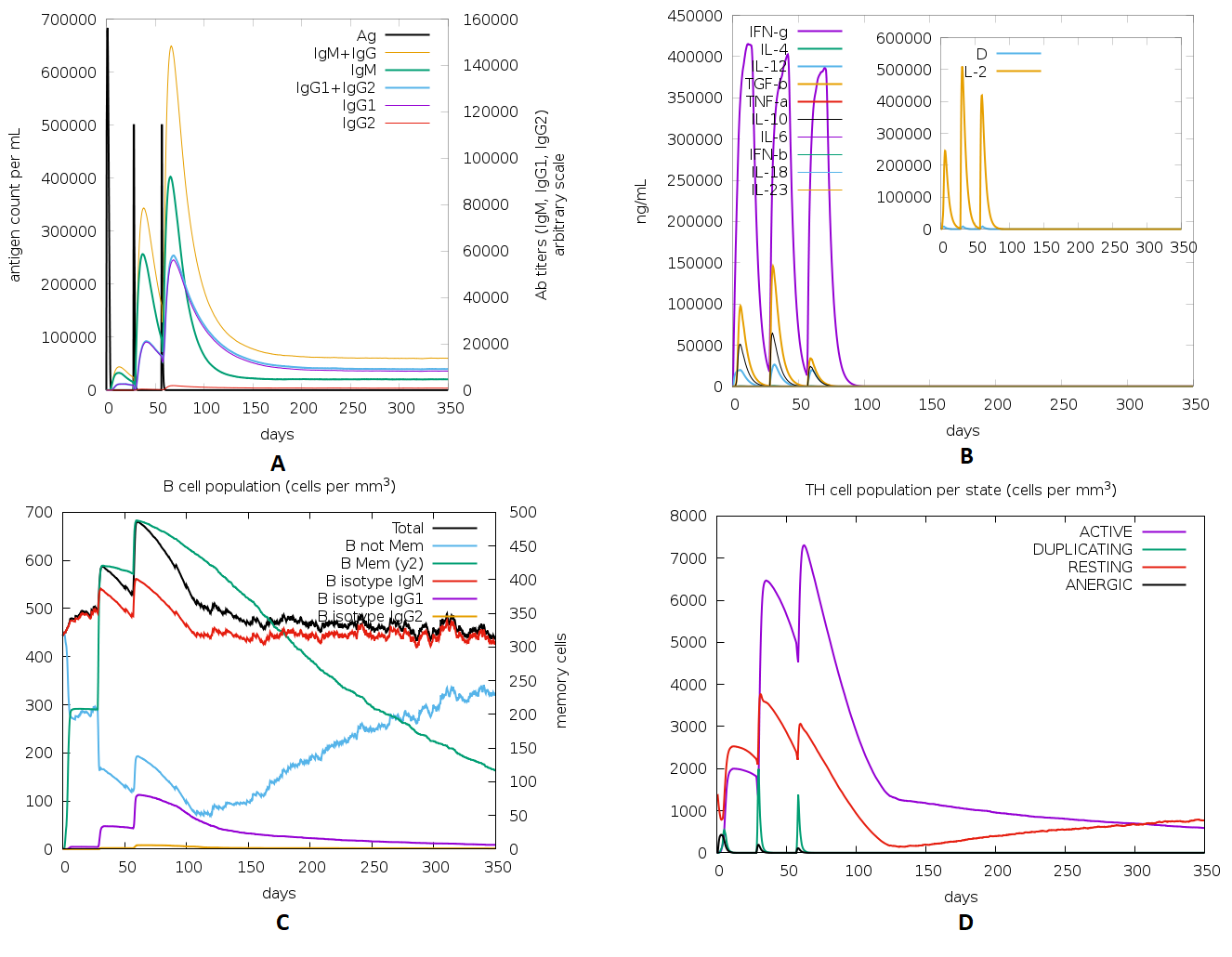


**Supplementary figure4.** Immune response predicted through ImmSim server after the injection of the designed multitope vaccine. (A) Levels of the produced antibodies. (B) Cytokines level, (C,D) demonstrate the B and T cell population respectively.
